# SUMO Modification of Hepatitis B Virus Core Mediates Nuclear Entry, Promyelocytic Leukemia Nuclear Body Association, and Efficient Formation of Covalently Closed Circular DNA

**DOI:** 10.1128/spectrum.00446-23

**Published:** 2023-05-18

**Authors:** Samuel Hofmann, Verena Plank, Peter Groitl, Nathalie Skvorc, Katharina Hofmann, Julius Luther, Chunkyu Ko, Peter Zimmerman, Volker Bruss, Daniela Stadler, Arnaud Carpentier, Shahinda Rezk, Michael Nassal, Ulrike Protzer, Sabrina Schreiner

**Affiliations:** a Institute of Virology, School of Medicine, Technical University of Munich, Germany; b Institute of Virology, Hannover Medical School, Hannover, Germany; c Institute of Virology, Helmholtz Zentrum München, Munich, Germany; d Department of Internal Medicine II/Molecular Biology, University Hospital Freiburg, Freiburg, Germany; e Institute of Experimental Virology, Twincore, Hannover, Germany; f Medical Research Institute, Department of Molecular and Diagnostic Microbiology, Alexandria University, Alexandria, Egypt; g German Center for Infection Research, Munich, Germany; h Cluster of Excellence RESIST (Resolving Infection Susceptibility; EXC 2155), Hannover Medical School, Hannover, Germany; University of Manitoba

**Keywords:** HBV, chronic infection, HBV core protein, SUMO, PML-NBs

## Abstract

Persistence of hepatitis B virus (HBV) infection is due to a nuclear covalently closed circular DNA (cccDNA), generated from the virion-borne relaxed circular DNA (rcDNA) genome in a process likely involving numerous cell factors from the host DNA damage response (DDR). The HBV core protein mediates rcDNA transport to the nucleus and likely affects stability and transcriptional activity of cccDNA. Our study aimed at investigating the role of HBV core protein and its posttranslational modification (PTM) with SUMO (small ubiquitin-like modifiers) during the establishment of cccDNA. HBV core protein SUMO PTM was analyzed in His-SUMO-overexpressing cell lines. The impact of HBV core SUMOylation on association with cellular interaction partners and on the HBV life cycle was determined using SUMOylation-deficient mutants of the HBV core protein. Here, we show that the HBV core protein is posttranslationally modified by the addition of SUMO and that this modification impacts nuclear import of rcDNA. By using SUMOylation-deficient HBV core mutants, we show that SUMO modification is a prerequisite for the association with specific promyelocytic leukemia nuclear bodies (PML-NBs) and regulates the conversion of rcDNA to cccDNA. By *in vitro* SUMOylation of HBV core, we obtained evidence that SUMOylation triggers nucleocapsid disassembly, providing novel insights into the nuclear import process of rcDNA. HBV core protein SUMOylation and subsequent association with PML bodies in the nucleus constitute a key step in the conversion of HBV rcDNA to cccDNA and therefore a promising target for inhibiting formation of the HBV persistence reservoir.

**IMPORTANCE** HBV cccDNA is formed from the incomplete rcDNA involving several host DDR proteins. The exact process and the site of cccDNA formation are poorly understood. Here, we show that HBV core protein SUMO modification is a novel PTM regulating the function of HBV core. A minor specific fraction of the HBV core protein resides with PML-NBs in the nuclear matrix. SUMO modification of HBV core protein mediates its recruitment to specific PML-NBs within the host cell. Within HBV nucleocapsids, SUMOylation of HBV core induces HBV capsid disassembly and is a prerequisite for nuclear entry of HBV core. SUMO HBV core protein association with PML-NBs is crucial for efficient conversion of rcDNA to cccDNA and for the establishment of the viral persistence reservoir. HBV core protein SUMO modification and the subsequent association with PML-NBs might constitute a potential novel target in the development of drugs targeting the cccDNA.

## INTRODUCTION

Hepatitis B virus (HBV) is a major global health threat, with two billion people having markers of previous infection and over 250 million being chronic virus carriers. HBV is a prototypic member of the family *Hepadnaviridae* with a small (3.2-kb), partially double-stranded (ds) relaxed circular DNA (rcDNA) genome, packaged in an icosahedral capsid consisting mostly of 240 core protein subunits. Upon entry into the host cell, the rcDNA-containing nucleocapsid is transported to the nuclear pore complex, where disassembly is triggered by a poorly understood mechanism. rcDNA subsequently enters the nucleus and is repaired into the ds plasmid-like covalently closed circular DNA (cccDNA). Several host DDR proteins have been found to participate in this conversion, but the full complement of involved host factors is likely much larger (reviewed in references 1 and 2). More recently, using a model involving synthetic rcDNA and cell extracts depleted of specific DDR factors, Wei and Ploss identified five core factors—polymerase δ, FEN1, DNA ligase 1, proliferating cell nuclear antigen (PCNA), and the replication factor C complex (RFC)—as both necessary and sufficient to promote cccDNA formation (3). By association with host histone and nonhistone proteins, cccDNA becomes chromatinized and then can serve as the template for transcription of viral mRNAs; transcriptional activity is subject to epigenetic regulation by the host, which in turn is modulated by the viral HBV core and HBx (reviewed in references 1, 4, and 5).

HBV core is a dimeric 21-kDa phosphoprotein, consisting of 183 or 185 amino acids, depending on the HBV genotype. Beyond its obvious role as a building block of the capsid, HBV core is an important mediator of almost every step in the HBV life cycle (6, 7). In the nucleus, it reportedly interacts with cellular chromatin, modulating transcription in the infected hepatocyte (7–9). The core protein primary sequence can be divided into an N-terminal assembly domain (NTD) of 140 amino acids and an arginine-rich C-terminal domain (CTD [or ARD]), connected through a flexible linker (10). Several studies indicate that HBV core is subject to extensive posttranslational modification (PTM) (reviewed in references 4, 10, and 11) and that it associates with promyleocytic leukemia nuclear bodies (PML-NBs) in HBV producing cell lines and under cellular stress induced by DNA damage (12). However, the molecular mechanism and biological consequences of this interaction during HBV infection are not understood. An additional interplay of PML-associated proteins and HBV enhancing viral replication is the association of the PML-resident 110-kDa speckled protein (Sp110) with HBx to promote HBV transcriptional activity (13).

PML-NBs are highly dynamic multiprotein complexes residing within the nuclear matrix. They appear as characteristic nuclear dots with an average size of 0.2 to 1 μm and a frequency of 2 to 30 bodies per cell (14). Besides the PML scaffold protein, other constitutive components of the bodies are Sp100, Daxx, and SUMO proteins (15). Depending on cell type, cell cycle, and stress status, a large variety of further proteins can be recruited to PML-NBs, preferentially via interaction of SUMO-interacting motifs (SIM) with SUMOylated proteins (14). PML-NBs can therefore be seen as model systems for phase separation in liquid-liquid interfaces, in which the scaffold is built up by RBCC-mediated interactions between PML proteins, as well as SUMO-SIM interactions within the PML scaffold. PML-NB composition is then further regulated by the SUMO status of the PML proteins. A low-SUMO PML scaffold recruits interactor proteins which are SUMOylated, while a high-SUMO PML scaffold mainly interacts with proteins harboring a SIM. PML-NB constitution is therefore mainly governed by the dynamic SUMOylation and deSUMOylation of the PML-NB scaffold (16–18). These SUMOylation hot spots of the cell are essential and dynamic mediators in many cellular regulation processes, like gene transcription and chromatin dynamics, cell growth, apoptosis and senescence, regulation of the immune response, protein degradation, and PTMs (19–21). PML-NBs are important hubs in the DNA damage response (DDR), accumulating at sites of DNA damage and recruiting important DDR factors often by SUMO/SIM association (22). It is therefore noteworthy that several of the above-mentioned core factors necessary for cccDNA formation discovered by Wei and Ploss (3) are SUMO modified and associate with PML protein to promote their function, including FEN1 (23–26), DNA ligase 1 (3, 26, 27), and PCNA (28–35). SUMO PTM occurs via a mechanism very similar to that of ubiquitinylation. SUMO chains are usually attached to lysine residues within so-called SUMO conjugation motifs (SCMs) encoded by the sequence ψKxE/D (with ψ being a hydrophobic and x any amino acid residue), a process mediated by SUMO E3 ligases. However, SUMO ligation of lysine residues can also occur in noncanonical SCMs. SUMOylation is involved in various signaling processes and also in the regulation of protein stability, conformation, and interaction potential and thus in activity and subcellular localization, in particular, recruitment to PML-NBs (36). DNA virus genomes and replication sites are often observed juxtaposed to PML-NBs, and various functions of viral proteins are extensively dependent on SUMO PTM by either host SUMOylation enzymes or viral surrogates (37).

Here, we show that the HBV core represents a so-far-unrecognized substrate for SUMOylation. HBV core is conjugated with SUMO moieties at two distinct SCMs within the viral protein that are highly conserved among all HBV genotypes. Though only a small portion of HBV core is SUMOylated, this particular fraction localizes in the nonsoluble nuclear matrix fraction, indicating that it is recruited into specific, but not all, PML-NBs during infection. SCM mutations diminished colocalization and interaction of HBV core with PML protein during transfection and infection, concomitant with a deficiency of HBV nucleocapsids in nuclear entry, which impaired cccDNA formation. Based on *in silico* structural prediction of SUMO2 docking onto core subunits in the HBV capsid and *in vitro* SUMOylation of purified recombinant HBV capsids, we propose that HBV core SUMOylation efficiently contributes to disassembly of the nucleocapsid within the nuclear basket of the nuclear pore complex. Our studies suggest a new model for the nuclear entry process of HBV, in which disruption of the nucleocapsid at the nuclear pore complex is initiated by HBV core SUMOylation in the capsid. We further observe the corecruitment of the SUMOylated HBV core fraction and HBV DNA to PML-NBs, which we propose as the sites where cccDNA generation takes place, mediated by regulatory host DDR proteins. Based on these findings, interference with the HBV core SUMOylation should provide a novel intervention strategy targeting a critical step in the HBV life cycle.

## RESULTS

### HBV core protein represents a novel substrate for the host cell SUMOylation machinery.

**FIG 1:**
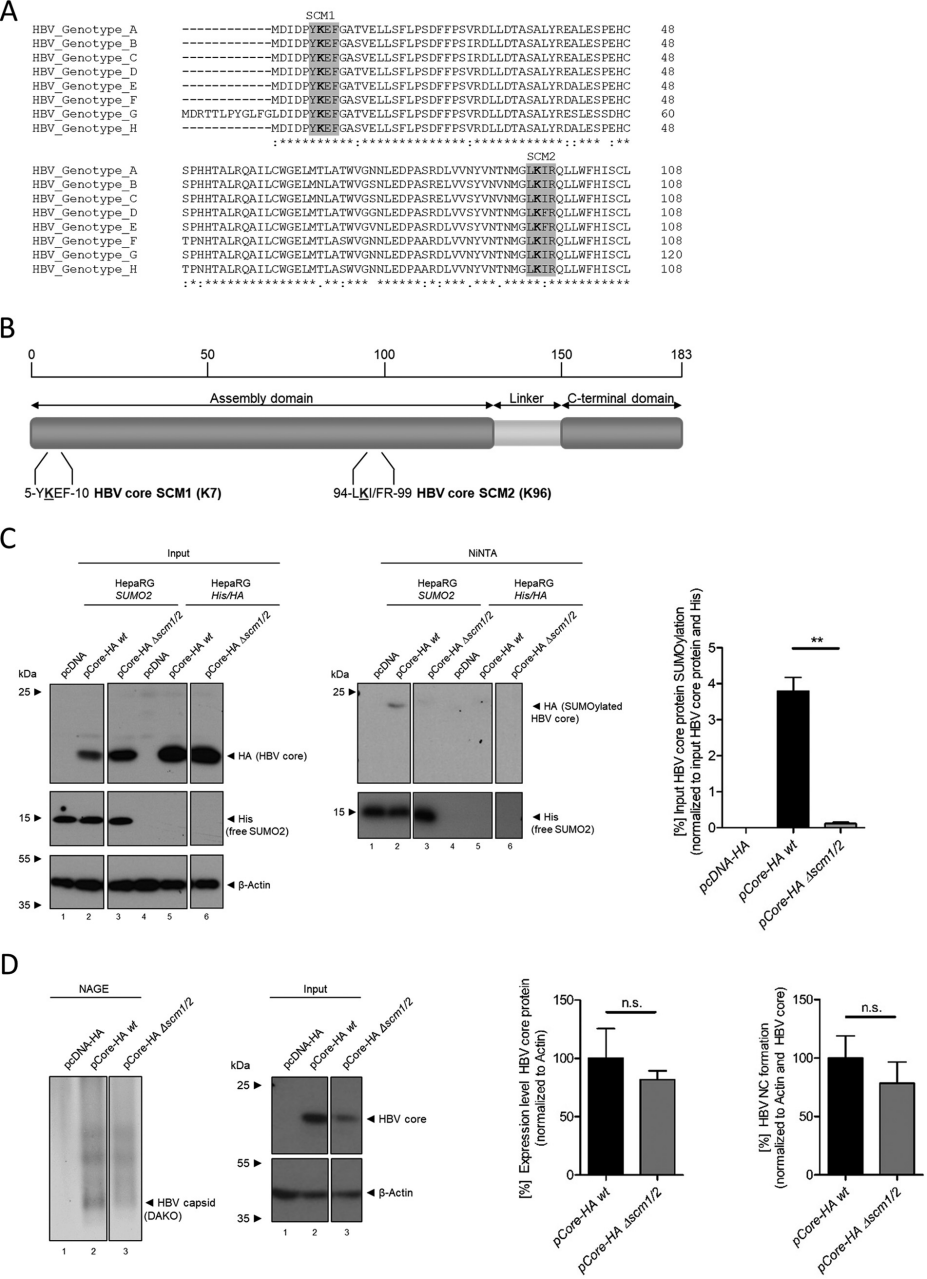
HBV core protein is SUMO modified at highly conserved SUMO conjugation motifs. (A) Consensus coding sequences for the HBV core protein of the HBV genotypes A to H were downloaded from HBVdb (78) and analyzed by multiple-sequence alignment using the Clustal Omega online tool (80). SUMO conjugation motifs (SCMs) within the HBV core protein coding sequences were predicted using GPS-SUMO 1.0 (79, 86). The highly conserved SCM1 and SCM2 are highlighted in gray. (B) Schematic representation of the HBV core protein amino acid sequence, consisting of the N-terminal assembly domain (amino acids [aa] 1 to 140) and the CTD (aa 150 to 183). Both SCMs are indicated. (C) HepaRG cells stably overexpressing His_6_-tagged SUMO2 and HepaRG cells stably expressing His_6_-HA as controls were transfected with 10 μg of pCore-HA wt and a mutant lacking SCM1 and SCM2 (pCore-HA Δ*scm1/2*) and harvested 48 h posttransfection. The samples were lysed using a denaturing guanidine hydrochloride buffer, and His_6_-SUMO2 modified proteins were isolated using a Ni-NTA pulldown. Ni-NTA-purified proteins and input whole-cell lysates were separated by SDS-PAGE and subjected to immunoblotting. The proteins of interest were detected using MAb 3F10 (anti-HA), MAb 6His (anti-6His-tag), and MAb AC-15 (anti-beta-actin). Levels of HBV core SUMO2 modification normalized to input levels and SUMO2 pulldown were determined using Fiji (version 1.45s). The bar chart shows average values and standard deviations calculated from two independent experiments. Statistical significance was determined using a two-sided Welch’s *t* test. **, *P* ≤ 0.01. (D) HepaRG cells were transfected with 5 μg of pCore-HA wt or pCore-HA Δ*scm1/2* and harvested 48 h posttransfection. The samples were lysed by addition of a low-stringency NP-40 lysis buffer that retains the native structure of the HBV nucleocapsids. Native lysates were subjected to NAGE and analyzed by immunoblotting using PAb B0586 (anti-HBV capsid). Steady-state levels of proteins were determined by denaturation of the same samples using Laemmli buffer and analysis by SDS-PAGE and immunoblotting using MAb 8C9-11 (anti-core) and MAb AC-15 (anti-beta-actin). Intensity of the respective protein bands was determined using Fiji (version 1.45s) in two independent experiments. Steady-state levels of HBV core protein were normalized to the wt protein, and anti-beta-actin. HBV nucleocapsid levels were normalized to the wt protein as well as the input expression level and anti-beta-actin. Mean values and standard deviations are presented in the bar charts. Statistical significance was determined using a two-sided Welch’s *t* test. n.s., not significant.

HBV core is subject to a large variety of posttranslational modifications governing the different functions that core can take over during the HBV life cycle (reviewed in reference 4). As previous studies showed HBV core binding to PML protein in cell lines with a stably integrated HBV genome (12), and as most proteins are recruited into PML-NBs via SUMO-SIM interactions (14, 38), we performed an *in silico* prediction for SCMs within the HBV core protein sequence utilizing the program GPS SUMO1.0. Both HBV core lysine residues, K7 and K96, matched the SCM consensus (SCM1 and SCM2), and both are highly conserved among all eight major HBV genotypes, with 99.869% conservation for K7 and 99.637% for K96 (Fig. 1A).

To confirm the SUMO modification of HBV core and the identity of the SCMs, the coding sequence of a genotype D HBV core was cloned into a pcDNA3.1-HA vector to allow expression of an N-terminally hemagglutinin (HA)-tagged core protein (pCore-HA wt). Residues K7 and K96 were replaced with arginines (pCore-HA Δ*scm1/2*) (Fig. 1B); HepaRG *SUMO2* cells (stably overexpressing His-tagged SUMO2 protein) and HepaRG *His/HA* (as control cells to exclude unspecific binding of HBV core) were transfected with expression vectors for HA-tagged wild-type (wt) HBV core protein or the Δ*scm1/2* mutant. Lysates of transfected cells or control cells were subjected to nickel nitrilotriacetic acid (Ni-NTA) pulldown. Results revealed an association of HBV core with SUMO2, which was completely lost in the Δ*scm1/2* HBV core (Fig. 1C, lanes 2 and 3). To prove that the HA-tagged HBV core protein produced after transfection of pCore-HA wt or pCore-HA Δ*scm1/2* was still capable of forming nucleocapsid structures, native agarose gel electrophoresis was performed. The results showed that neither the N-terminal HA-tag nor mutation of SCM1 and SCM2 interfered with efficient formation of nucleocapsids (Fig. 1D, lanes 2 and 3). Hence, HBV core K7 and K96 can be targets for SUMO modification, and arginine mutation of these lysine residues abrogates SUMOylation of the viral protein without affecting the ability to form nucleocapsids.

### HBV core SUMOylation is a prerequisite for association with PML-NBs.

**FIG 2:**
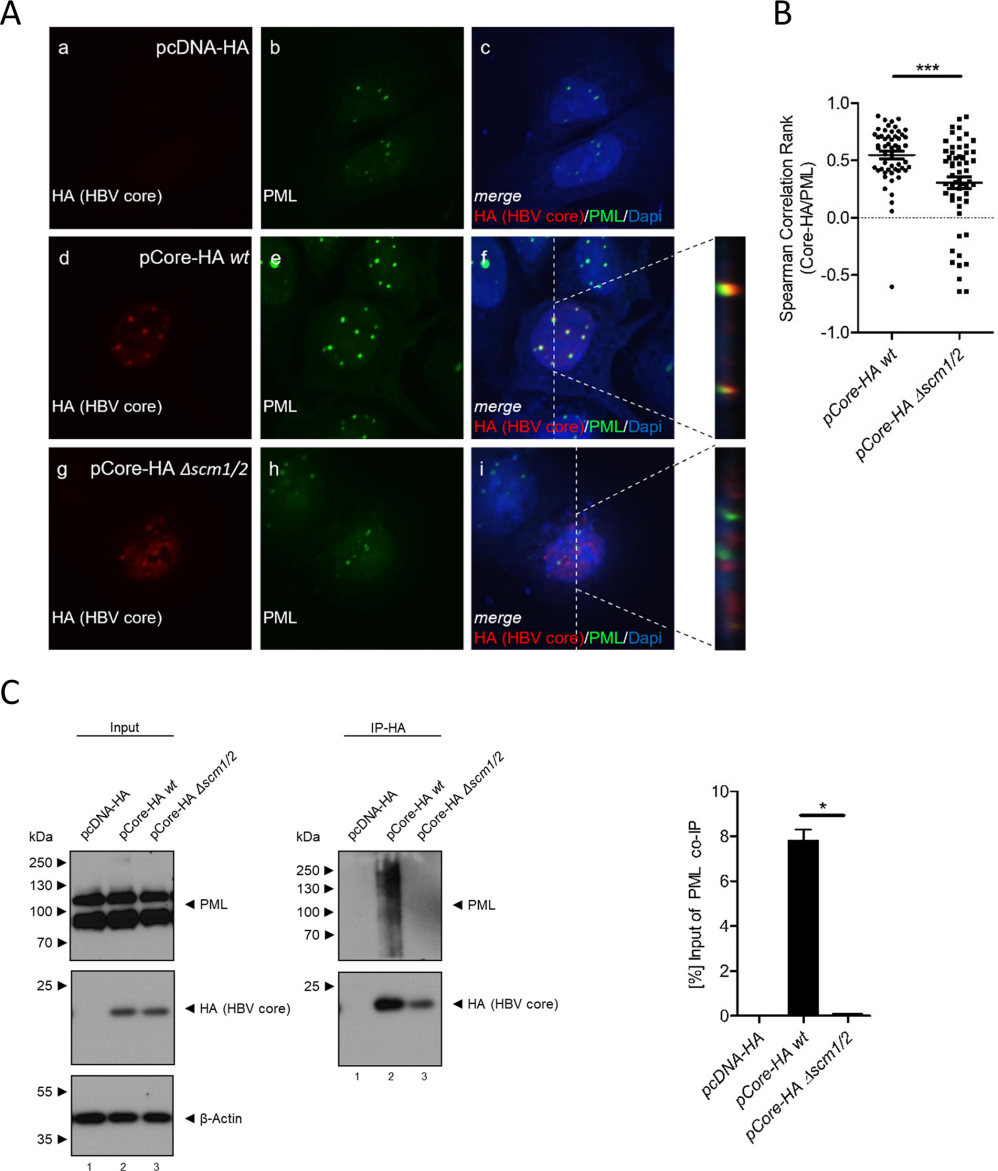
HBV core protein SUMO modification is a prerequisite for its association with PML protein. (A) HepaRG cells were transfected with 10 μg of pCore-HA wt (panels d to f) or Δ*scm1/2* (panels g to i) and fixed with 4% PFA 48 h posttransfection. PML protein and the HA tag of the HBV core protein were detected by indirect immunofluorescence using PAb NB100-59787 (anti-PML protein) and MAb 3F10 (anti-HA). The primary antibodies were detected using Alexa Fluor 647 (HA; red)- and Alexa Fluor 488 (PML protein; green)-coupled secondary antibodies. Images and Z-stacks were taken using a Nikon TiE microscope equipped with the Perkin Elmer UltraView Vox system. Images show the staining patterns of at least 40 cells. (B) Spearman correlation rank of HBV core and PML protein signals was determined for at least 40 cells using Fiji software and the Pearson-Spearman correlation colocalization plug-in. Statistical significance was determined using one-way ANOVA and Dunn’s posttest. ***, *P* ≤ 0.001. (C) HepaRG cells were transfected with 10 μg of pCore-HA wt and pCore-HA Δ*scm1/2*. Total-cell protein lysates were prepared 48 h posttransfection. Immunoprecipitation of HA was performed using monoclonal >rat anti-HA hybridoma supernatant 3F10 (anti-HA). Proteins from total-cell protein lysates, as well as from the immunoprecipitation experiment were separated by SDS-PAGE and subjected to immunoblotting using PAb NB100-59787 (anti-PML protein), MAb 3F10 (anti-HA), and MAb AC-15 (anti-beta-actin). Western blot signals were quantified using densitometric analysis with Fiji (version 1.45s). Signals for coprecipitated PML protein were calculated as percentage of input of PML protein co-IP. Bar charts show average values and standard deviations calculated from two independent experiments. Statistical significance was determined using a two-sided Welch’s *t* test. *, *P* ≤ 0.05.

To assess whether HBV core interacts with PML protein independently of other viral factors and to determine the possible impact of HBV core SUMOylation on the association with PML-NBs, plasmids encoding HA-tagged wt or Δ*scm1/2* HBV core were transfected into HepaRG cells. Intracellular immunofluorescence analyses showed a distinct and almost exclusive colocalization of the wt HBV core with PML-NBs (Fig. 2A, panels d to f). In line with the loss of SUMO modification in the Δ*scm1/2* HBV core (Fig. 1C), this colocalization was no longer detectable in the double mutant (Fig. 2A, panels g to i). Spearman correlation rank analysis of the colocalization of HBV core and PML protein further confirmed a significant decrease in HBV core-PML protein association for Δ*scm1/2* HBV core compared to the wt (Fig. 2B).

For further confirmation, HepaRG cells were transfected with plasmids encoding either HA-tagged wt or Δ*scm1/2* HBV core, and total-cell lysates were subjected to coimmunoprecipitation assays using an anti-HA antibody. While wt HBV core coprecipitated PML protein (Fig. 2C, lane 2), confirming an interaction between wt HBV core and PML protein during transfection, Δ*scm1/2* HBV core had lost this ability (Fig. 2C, lane 3). Thus, PML protein did not run as completely clear bands but showed a smeary running behavior, indicative of posttranslational modification of the coprecipitated PML proteins (39). This strongly suggests that HBV core SUMOylation is the prerequisite to associate with PML-NBs.

### A specific minor fraction of HBV core colocalizes with PML protein during HBV infection.

**FIG 3:**
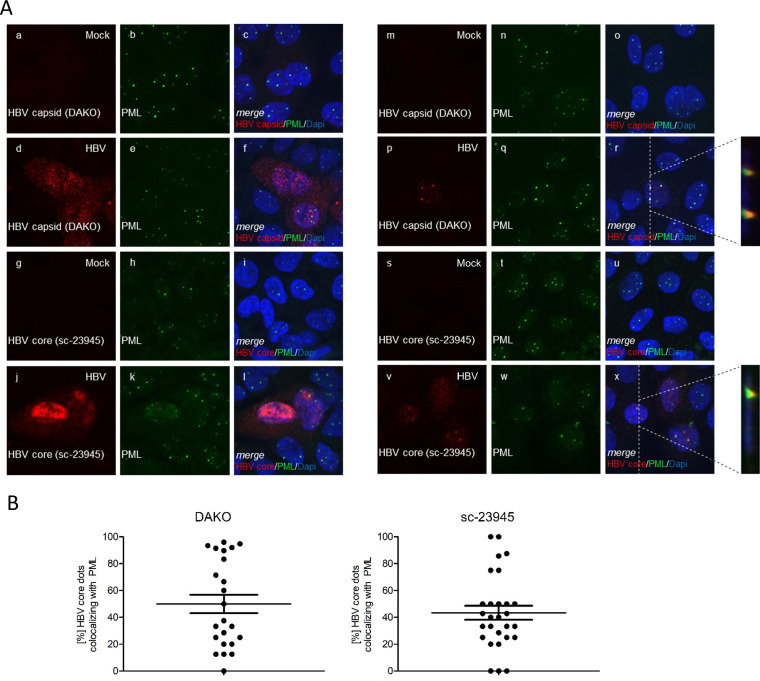
A minor specific fraction of HBV core protein colocalizes with PML protein during infection. (A and B) HepG2-NTCP-K7 cells were differentiated in the presence of 2.5% DMSO for 3 days and infected with HBV at an MOI of 400. Cells were fixed with 4% PFA (panels a to l) or treated with CSK buffer for washout of soluble proteins prior to fixation (panels m to x) 7 days postinfection and double labeled with PAb B0586 (anti-HBV capsid) and MAb sc-966 (anti-PML protein) (panels a to f and m to r) or MAb sc-23945 (anti-HBV core) and PAb NB100-59787 (anti-PML protein) (panels g to l and s to x). Primary antibodies were labeled with Alexa Fluor 488 (PML protein; green)- and Alexa Fluor 647 (HBV capsid; red)-conjugated secondary antibodies Images and Z-stacks were taken using a Nikon TiE microscope equipped with the Perkin Elmer UltraView Vox system. Representative staining for at least 30 mock-infected or HBV-infected cells, as indicated, is shown in panels a to l, and at least 20 mock-infected or infected cells, as indicated, with the CSK wash are shown in panels m to x. (B) HBV core protein signals colocalizing with PML protein were quantified from at least 20 infected cells with CSK wash stained with PAb B0586 (anti-HBV capsid [Dako]) (left) or stained with MAb sc-23945 (anti-HBV core) (right) and visualized as percentage of HBV core protein dots colocalizing with PML protein.

Previous publications reported a diffuse nuclear and cytoplasmic distribution of HBV core during infection (40, 41). HBV infection of differentiated HepG2-NTCP-K7 cells confirmed this diffuse phenotype using an antibody against assembled HBV capsid/capsid intermediate structures (Fig. 3A, panels d to f) and an antibody targeting a linear epitope within HBV core (Fig. 3A, panels j to l).

Addition of a cytoskeleton (CSK) buffer removes any soluble protein fraction from the cells and leaves only the insoluble protein fraction, including proteins of the nuclear matrix, such as PML protein (42). CSK treatment made distinct nuclear speckles of the HBV core protein visible, and they efficiently colocalized with some specific PML-NBs using antibodies detecting either assembled HBV capsids or nondenatured capsid assembly intermediates (43) (Fig. 3A, panels p to r) or the antibody directed against a linear epitope within the HBV core protein between amino acids 74 and 89 (44) (Fig. 3A, panels v to x). Thus, our findings confirm an association of a specific minor fraction of the HBV core with PML-NBs during HBV infection of hepatocytes. Here, HBV core was observed to localize to a median of 50% of PML-NBs in infected cells using either an antibody against assembled HBV capsid structures (Fig. 3B, left) or an antibody targeting a linear epitope within HBV core (Fig. 3B, right), indicating recruitment to specific, but not all, PML-NBs.

### HBV core SUMOylation promotes cccDNA formation.

**FIG 4:**
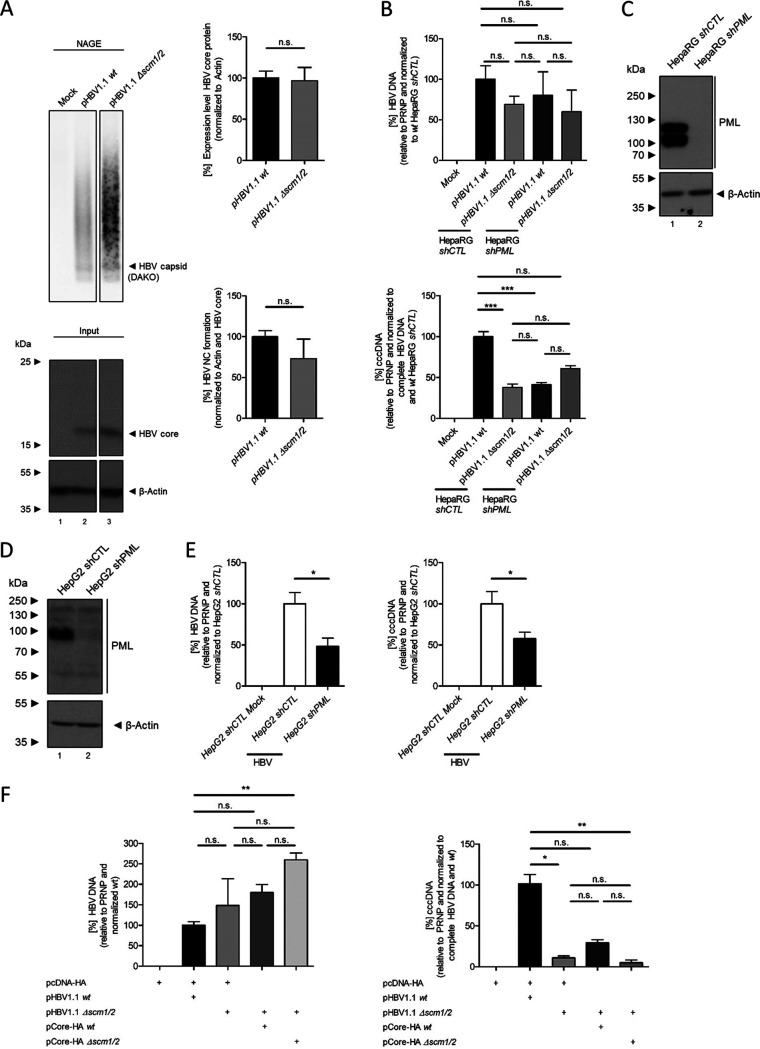
HBV core protein SUMO PTM is necessary for efficient cccDNA formation. (A) HepaRG cells were transfected with 5 μg of pHBV1.1 wt (lane 2) or Δ*scm1/2* (lane 3) and harvested 7 days posttransfection. The samples were lysed by addition of a low-stringency NP-40 lysis buffer that retains the native structure of the HBV nucleocapsids. Native lysates were subjected to NAGE and analyzed by immunoblotting using PAb B0586 (anti-HBV capsid). Steady-state levels of proteins were determined by denaturation of the same samples using Laemmli buffer and analysis by SDS-PAGE and immunoblotting using MAb 8C9-11 (anti-core) and MAb AC-15 (anti-beta-actin). Intensity of the respective protein bands was determined using Fiji (version 1.45s) in three independent experiments. Steady-state levels of HBV core protein were normalized to the wt protein and anti-beta-actin. HBV nucleocapsid levels were normalized to the wt protein, as well as the input expression level and anti-beta-actin. Mean values and standard deviations are presented in the bar chart. (B) HepaRG cells with stable transduction of a scrambled control shRNA (HepaRG shCTL), or a shRNA against a conserved region in PML protein (HepaRG shPML) were transfected with 3 μg pHBV1.1 wt or pHBV1.1 Δ*scm1/2*. Total DNA of the samples was isolated, and total HBV DNA (top) and cccDNA (bottom) were measured 7 days posttransfection by qPCR relative to prion protein P (PRNP). cccDNA levels were normalized to the levels of total HBV DNA. Bar charts present mean values and standard deviations from six independent experiments measured in triplicate. Statistically significant differences were determined using one-way ANOVA. n.s., not significant; ***, *P* ≤ 0.001 (C and D) HepaRG (C) and HepG2-NTCP-K7 (D) cells were transduced with lentiviral vectors encoding either a scrambled control shRNA (HepaRG/HepG2 shCTL) or a shRNA directed against PML protein (HepaRG/HepG2 shPML). Total-cell protein lysates of HepaRG shCTL and HepaRG shPML (C) or HepG2 shCTL and HepG2 shPML (D) cells were prepared, separated by SDS-PAGE, and subjected to immunoblotting using PAb NB100-59787 (anti-PML protein) and MAb AC-15 (anti-beta-actin). (E) HepG2-NTCP cells stably transduced with a control shRNA (HepG2 shCTL) or a shRNA targeting PML protein (HepG2 shPML) were differentiated using 2.5% DMSO for 3 days and infected with HBV at an MOI of 200. Seven days postinfection, total DNA was isolated, and total HBV DNA (left) and cccDNA (right) were determined by qPCR. The bar charts present values and standard deviations from six independent experiments measured in triplicate. Statistical significance was determined using a two-sided Welch’s *t* test. *, *P* ≤ 0.05. (F) HepaRG cells were transfected with 3 μg of pHBV1.1 wt or pHBV1.1 Δ*scm1/2* and 1 μg of pcDNA-HA, pCore-HA wt, or pCore-HA Δ*scm1/2*. Seven days posttransfection, total DNA was extracted and subjected to qPCR for total HBV DNA (left) and cccDNA (right). cccDNA levels were normalized to the levels of total HBV DNA. The bar charts present mean values and standard deviations from three independent experiments measured in duplicate. Statistically significant differences were determined using one-way ANOVA. n.s., not significant; *, *P* ≤ 0.05; **, *P* ≤ 0.01.

To further understand the functional consequences of HBV core SUMOylation on HBV replication, plasmids carrying 1.1 copies of the HBV genome under the control of the cytomegalovirus immediate early (CMV-IE) enhancer/promoter (pHBV1.1 wt) were used, and mutations of both (pHBV1.1 Δ*scm1/2*) SCMs within HBV core were introduced into the HBV genome. To address a potential disturbance of nucleocapsid formation by the SCM mutations, native lysates from transfected hepatocytes were subjected to native agarose gel electrophoresis (NAGE). Here, we detected wild type-like levels of core expression (Fig. 4A, top), as well as nucleocapsid formation (Fig. 4A, bottom).

To validate the impact of HBV core SUMOylation on the viral life cycle, HepaRG cells with short hairpin control RNA (shCTL) were transfected with plasmids pHBV1.1 wt or pHBV1.1 Δ*scm1/2* and examined for viral DNA formation as a marker for replication competence. While constitutive expression of pregenomic RNA (pgRNA) by a CMV-IE enhancer/promoter from the transfected plasmids should lead to strong formation of rcDNA, the minor cccDNA fraction should be established only upon nuclear import of newly formed rcDNA-containing nucleocapsids. Monitoring total HBV DNA amounts revealed only minor differences between wt and mutant HBVs (Fig. 4B, top), whereas cccDNA signals for Δ*scm1/2* (Fig. 4B, bottom) were significantly reduced when normalized to the levels of total HBV DNA, which is mostly rcDNA (45).

To further assess the importance of core-PML protein association for HBV replication, pHBV1.1 wt and pHBV1.1 Δ*scm1/2* were transfected into HepaRG shPML with a stable shRNA-mediated PML protein knockdown (Fig. 4C). Quantitative PCR (qPCR) analysis of total HBV DNA levels again showed only minor differences between the setups (Fig. 4B, top). Notably, PML protein depletion in HepaRG shPML cells correlated with similarly reduced cccDNA levels for wt HBV as observed for the Δ*scm1/2* mutant, indicating that a lack of PML protein evokes a phenotype similar to that seen with a lack of HBV core protein SUMOylation (Fig. 4B, bottom). To underline the importance of PML protein in efficient cccDNA formation, HepG2-NTCP-K7 cells depleted of PML protein (HepG2 shPML cells) (Fig. 4D) were infected with HBV, and total HBV DNA and cccDNA were monitored. Here, a similar phenotype was observed, where a loss of PML protein interfered with efficient HBV replication and cccDNA formation (Fig. 4E).

Next, we asked whether the reduced cccDNA phenotype of pHBV1.1 Δ*scm1/2* could be rescued in *trans* by wt HBV core. Indeed, coexpression of wt but not the variant HBV core increased cccDNA signals from pHBV1.1 Δ*scm1/2* (Fig. 4F, right), with cccDNA levels being up to 30% of those seen with wt pHBV1.1 when normalized to the levels of total HBV DNA, which is mostly rcDNA (45).

To validate the qPCR-mediated determination of cccDNA in pHBV1.1-transfected cells, samples were codigested with DpnI in addition to T5 exonuclease. Codigestion efficiently removed pHBV1.1 plasmid DNA diluted in DNA isolated from HepG2-NTCP-K7 cells (see Fig. S1B in the supplemental material) and therefore efficiently abrogated the detection of plasmid DNA in the qPCR assay, while leaving native cccDNA from an HBV infection untouched (Fig. S1C). The repetition of the qPCR determination of cccDNA for results shown in Fig. 4B and F showed the same patterns (Fig. S1D and E), confirming the presented results.

In conclusion, these data indicated a significant impact of HBV core SUMO modification and subsequent PML protein association on the establishment of the HBV cccDNA as the viral persistence reservoir.

### HBV core SUMOylation mediates nuclear entry and PML protein association during HBV infection.

**FIG 5:**
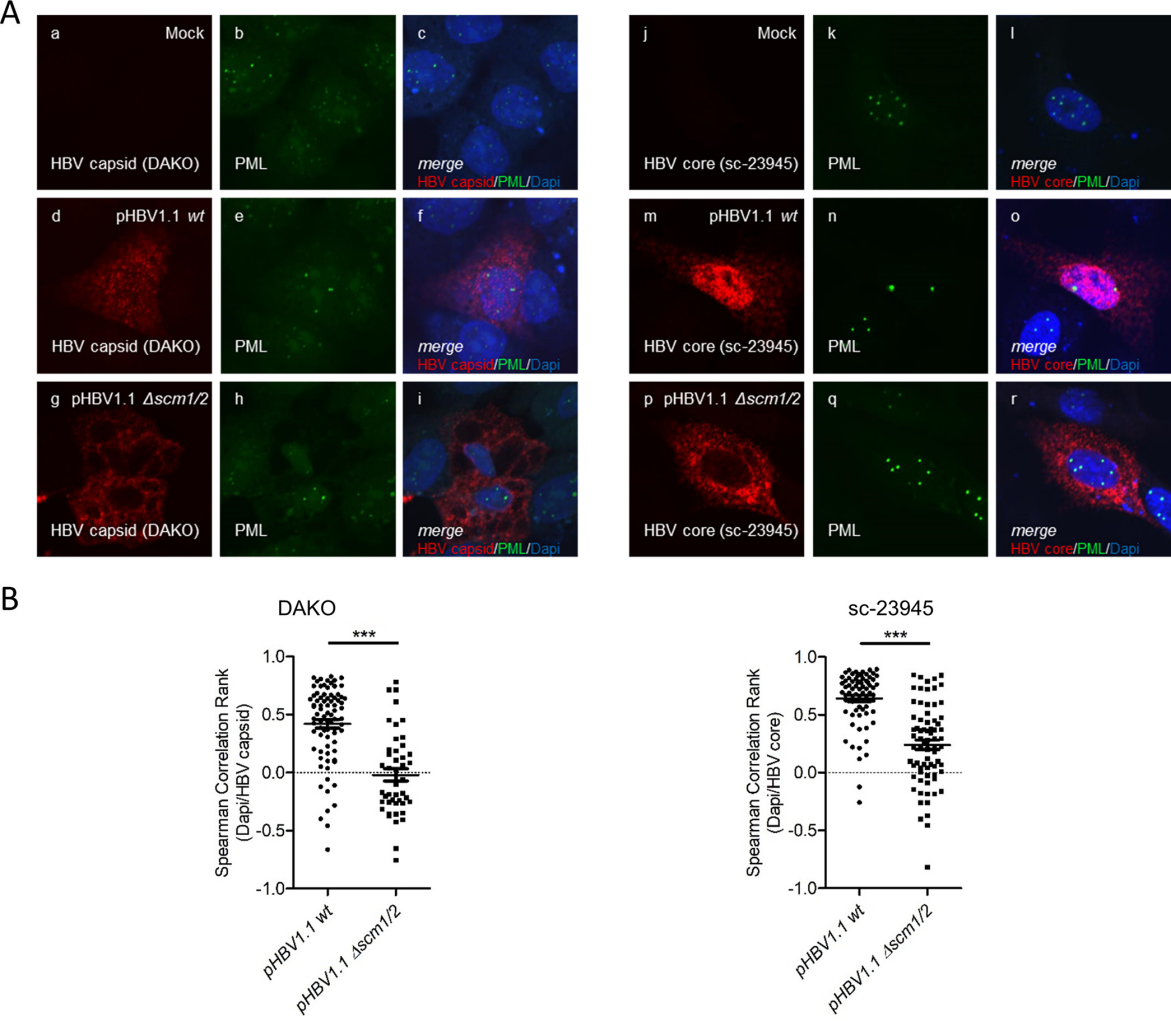
HBV core protein SUMO modification mediates nuclear localization during infection. (A and B) HepaRG cells were transfected with 3 μg pHBV1.1 wt (panels d to f and m to o) and pHBV1.1 Δs*cm1/2* (panels g to i and p to r) and fixed with 4% PFA 48 h posttransfection. HBV capsids and PML protein were double labeled with PAb B0586 (anti-HBV capsid) and MAb sc-966 (anti-PML protein) (panels a to i) or MAb sc-23945 (anti-HBV core) and PAb NB100-59787 (anti-PML protein) (panels j to r). Primary antibodies were detected with Alexa Fluor 488 (PML protein; green)- and Alexa Fluor 647 (HBV capsid/core; red)-conjugated secondary antibodies. Images were taken using a Nikon TiE microscope. (A) Representative staining of at least 45 cells is shown. (B) Colocalization of the HBV core protein or HBV capsid staining with the nucleus indicated by DAPI staining was determined using the Spearman correlation rank method and Fiji software with the Pearson-Spearman correlation colocalization plug-in. Statistical significance was determined using a two-sided Welch’s *t* test. ***, *P* ≤ 0.001.

To next investigate the impact of the SCM mutations on HBV core localization, pHBV1.1 wt and pHBV1.1 Δ*scm1/2* were transfected into HepaRG cells and subjected to intracellular immunofluorescence studies. For the wt HBV core, the diffuse intracellular distribution observed in infected cells (40, 41) was also observed with both antibodies, the one recognizing HBV nucleocapsids/capsid intermediates (Fig. 5A, panels d to f) and the one directed against a linear epitope (Fig. 5A, panels m to o). Importantly, the SUMOylation-deficient core (pHBV1.1 Δ*scm1/2*) localized almost exclusively outside the nucleus (Fig. 5A, panels g to i and p to r), as confirmed by measurement of the colocalization of HBV core with the nucleus using Spearman correlation rank (Fig. 5B).

**FIG 6:**
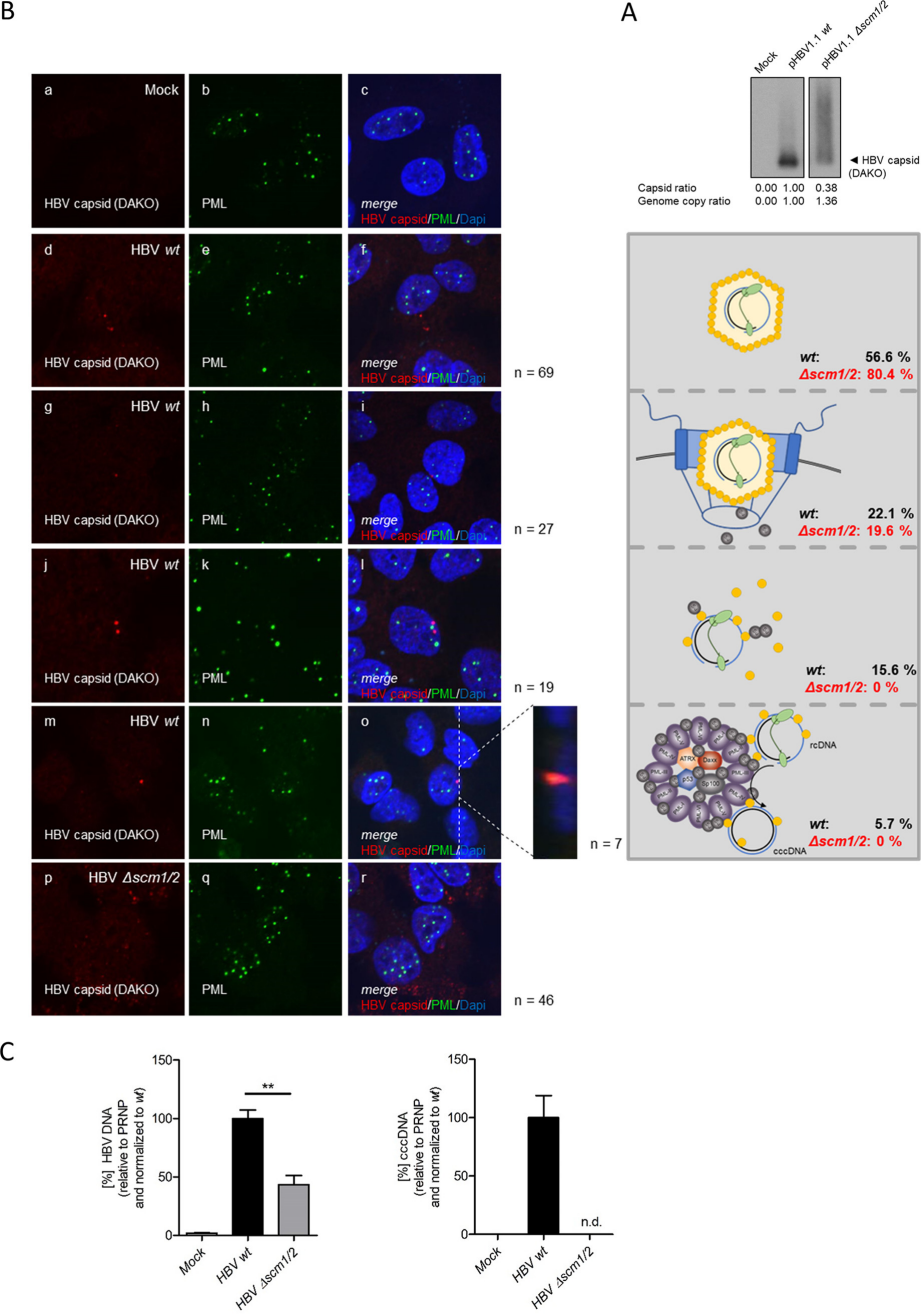
A minor specific proportion of entering HBV virions associates with PML-NBs. (A) HepaRG cells were transfected with 10 μg of pHBV1.1 wt or pHBV1.1 Δ*scm1/2*. Supernatants were harvested at days 3, 6, and 9 posttransfection, and HBV virions were isolated by PEG precipitation. The amount of secreted virions was assessed using NAGE and immunoblotting using PAb B0586 (anti-HBV capsid). Intensity of the respective protein bands in the NAGE was determined using Fiji (version 1.45s) and normalized to pHBV1.1 wt. Additionally, the genome copy number of the isolated virions per microliter was determined using qPCR and normalized to pHBV1.1 wt. (B) HepG2-NTCP-K7 cells were differentiated in the presence of 2.5% DMSO for 3 days and infected with HBV virions isolated from pHBV1.1 wt-transfected cells. At 24 hpi, cells were fixed with 4% PFA and double labeled with PAb B0586 (anti-HBV capsid) and MAb sc-966 (anti-PML protein). Primary antibodies were labeled with Alexa Fluor 488 (PML protein; green)- and Alexa Fluor 647 (HBV capsid; red)-conjugated secondary antibodies. Images and Z-stacks were taken using a Nikon TiE microscope equipped with the Perkin Elmer UltraView Vox system. Representative staining of at least 46 mock-infected, wt HBV-infected, or HBV Δ*scm1/2*-infected cells is shown in panels a to c, d to o, and p to r, respectively. The schematic representation shows the different hypothesized localizations of the HBV core protein during the entry process starting from a cytoplasmic localization (panels d to f) to localization at the border of the nuclear DAPI staining (panels g to i), transition to the nuclear lumen (panels j to l), and, ultimately, recruitment to PML-NBs (panels m to o). (C) HepG2-NTCP-K7 cells were differentiated in the presence of 2.5% DMSO for 3 days and infected with HBV virions isolated from pHBV1.1 wt- or pHBV1.1 Δ*scm1/2*-transfected cells at an MOI of 500. Total DNA was isolated 7 days postinfection, and total HBV DNA (left) and cccDNA (right) were determined by qPCR. The bar charts represent six independent experiments measured in triplicate. Statistically significant differences were determined using one-way ANOVA. **, *P* ≤ 0.01; n.d., not detectable.

Next, we transfected pHBV1.1 wt or pHBV1.1 Δ*scm1/2* into HepaRG cells and isolated infectious viral particles from the supernatants of transfected cells. Here, we observed secretion of infectious viral particles which also carried HBV DNA for both the pHBV1.1 wt-transfected and the pHBV1.1 Δ*scm1/2*-transfected HepaRG cells (Fig. 6A). The virions were used to infect differentiated HepG2-NTCP-K7 cells. Protein localization analyses of entering viral particles 24 h postinfection showed that in the context of viral replication, the respective HBV core mutations affect PML-NB colocalization and nuclear entry. During wt HBV infection, we observed that the majority (57%) of core was cytoplasmic (Fig. 6B, panels d to f), while 22% localized to the border of the nuclear 4′,6-diamidino-2-phenylindole (DAPI) staining (Fig. 6B, panels g to i). Sixteen percent of HBV core was recruited into the hepatocyte nucleus, without PML-NB association (Fig. 6B, panels j to l), and only a minor specific fraction of 6% was found juxtaposed to PML-NB SUMO hot spots in the cell (Fig. 6B, panels m to o). This is consistent with reports showing that during HBV infection, only 20% of the viral particles reach the host cell nucleus and 1% or even less produce cccDNA (46). In contrast, we observed that during infection with particles from pHBV1.1 Δ*scm1/2*-transfected cells, the cytoplasmic HBV core fraction was enhanced to 80%, while only 20% was found at the border of the nuclear DAPI staining; nothing could be detected in either the nuclear or the PML-NB fraction (Fig. 6B, panels p to r). Taken together, these results indicate that SUMOylation is critical to allow HBV core translocation to the nucleus during HBV infection.

To further assess the impact of lack of HBV core SUMO PTM and therefore loss of nuclear entry on the establishment of cccDNA, differentiated HepG2-NTCP-K7 cells were infected with the virus isolated from pHBV1.1 wt- or pHBV1.1 Δ*scm1/2*-transfected cells, and total HBV DNA and cccDNA were assessed 7 days postinfection. Here, a strong and significant reduction of total HBV DNA, including rcDNA and replication intermediates, was observed in cells infected with HBV Δ*scm1/2* compared to those infected with the wt (Fig. 6C, left), while cccDNA was undetectable in HBV Δ*scm1/2*-infected cells (Fig. 6C, right). Taken together, these findings indicated a strong impact of HBV core SUMOylation on nuclear entry and efficient formation of the HBV persistence reservoir, the cccDNA.

### HBV core SUMOylation triggers disassembly of HBV nucleocapsids.

**FIG 7:**
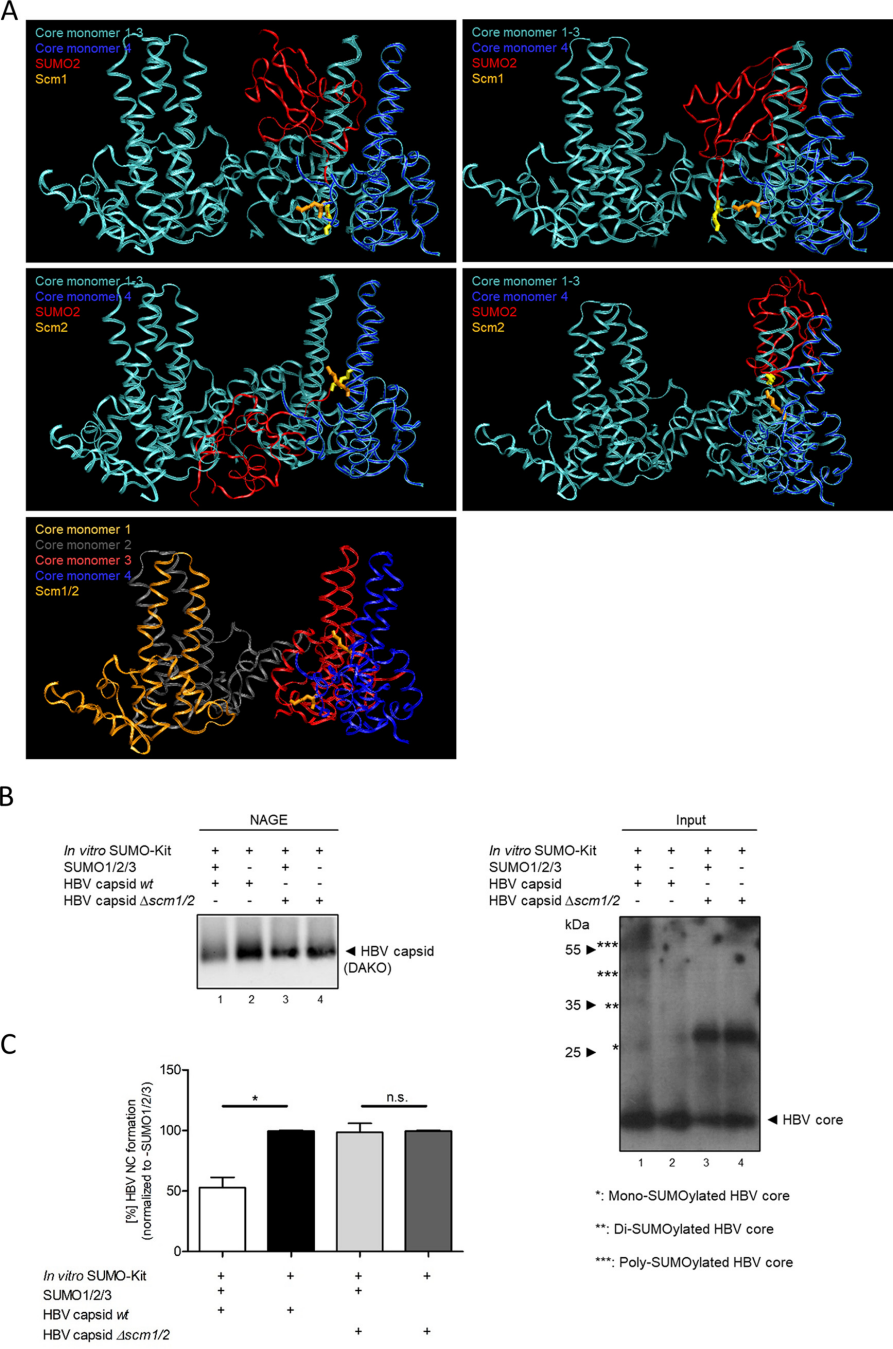
HBV core protein SUMOylation triggers HBV nucleocapsid disassembly. (A) Structures of the HBV core protein (PDB no. 3J2V_1) (81) and SUMO2 (PDB no. 2CHK_2) (82) were retrieved from the PDB and subjected to *in silico* docking analysis using the ZDOCK server (83). The predicted docking structures were visualized using VMD (84). The double-glycine motif at the C terminus of the SUMO2 protein is marked in yellow; SCM1 and SCM2 are in orange. (B) Recombinantly expressed wt and Δ*scm1/2* HBV capsid-like particles were subjected to an *in vitro* SUMOylation assay and further analyzed by NAGE (left) and SDS-PAGE (right) followed by immunoblotting using PAb B0586 (anti-HBV capsid) and MAb 8C9-11 (anticore), respectively. (C) The intensity of the respective protein bands in NAGE (panel B, left) was determined using Fiji (version 1.45s) in two independent experiments. Mean values and standard deviations are presented. Statistical significance was determined using a two-sided Welch’s *t* test. n.s., not significant; *, *P* ≤ 0.05.

As the HBV core Δ*scm1/2* variant showed an exclusive extranuclear phenotype in the context of viral replication in pHBV1.1 Δ*scm1/2*-transfected cells (Fig. 5A, panels g to i and p to r), and as virions derived from it were unable to enter the nucleus (Fig. 5C, panels m to o), the effect of HBV core protein SUMOylation on capsid stability was investigated. An *in silico* docking study of a dimer of HBV core protein dimers (the asymmetric unit of HBV T=4 and T=3 capsids) and SUMO2 revealed that the addition of a SUMO moiety (Fig. 7A, red) at either SCM1 or SCM2 (orange) caused a steric overlap with the contact site to the adjacent core protein (light blue). We thus hypothesized that SUMOylation of even a fraction of HBV core subunits per capsid could trigger disassembly.

To test this hypothesis, *in vitro* SUMOylation assays were performed with recombinant HBV capsid-like particles. Treatment with the purified SUMO machinery strongly reduced the wt capsid signal in the NAGE assay, indicating a loss of assembled HBV nucleocapsids (Fig. 7B, left, lane 1, and Fig. 7C), and additionally induced higher-migrating bands in the Western blot input (SDS-PAGE immunoblot), which indicates SUMO modification of HBV core within the capsid (Fig. 7B, right, lane 1). In contrast, Δ*scm1/2* HBV capsids were not affected by the *in vitro* SUMOylation machinery (Fig. 7B, lane 3, and Fig. 7C), indicating a SUMO-specific effect. For Δ*scm1/2* HBV capsids, higher-migrating bands could be detected above 30 kDa in samples with and without SUMO1/2/3, which might represent stable HBV core dimers (47), which occurred independently of the SUMO status of the protein (Fig. 7B, input panel, lanes 3 and 4). In conclusion, these results suggest that SUMOylation of HBV core subunits within the HBV capsids triggers capsid disassembly, providing a potential mechanism for rcDNA release into the nucleus.

### HBV DNA is recruited to PML-NBs.

**FIG 8:**
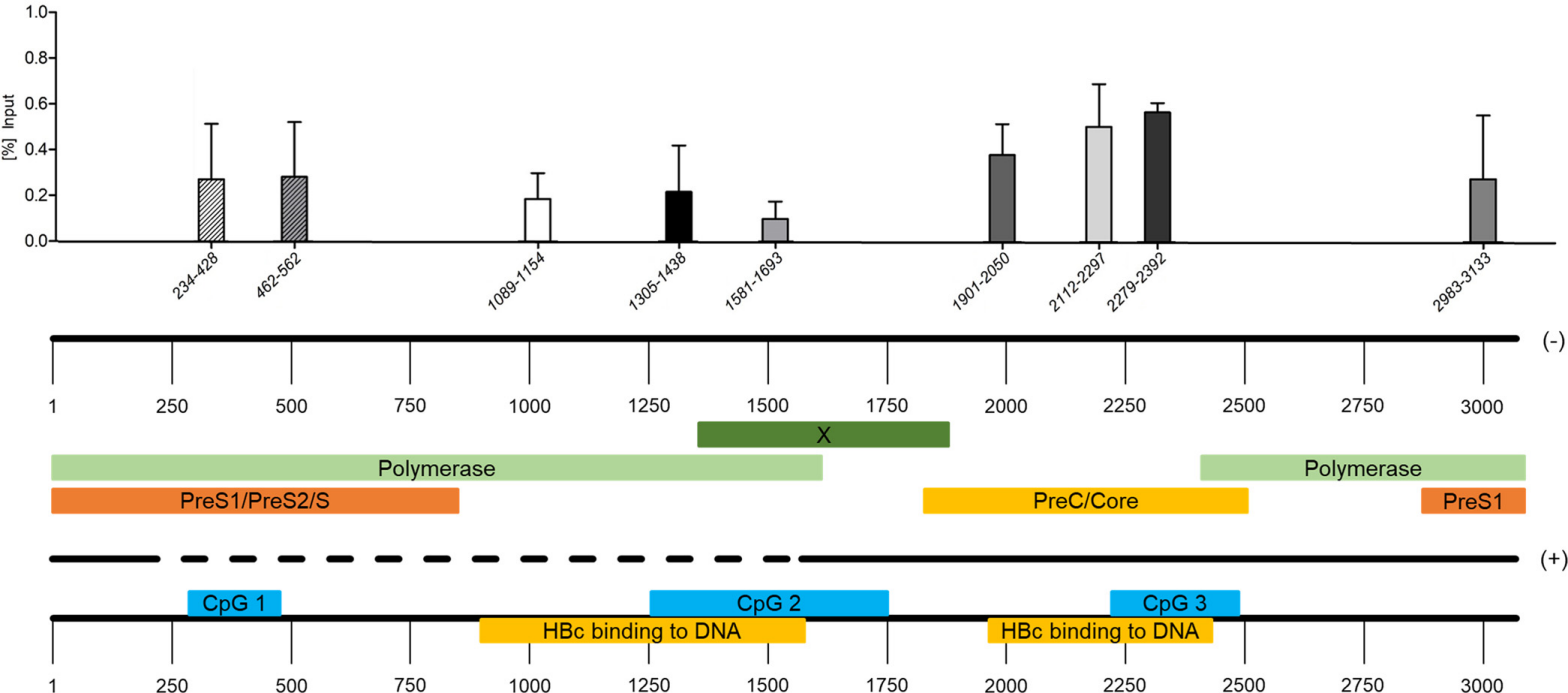
HBV DNA associates with PML-NBs. HepG2-NTCP-K7 cells were differentiated by addition of 2.5% DMSO for 3 days. Cells were infected with HBV at an MOI of 1,000. At 7 days postinfection, the cells were harvested and subjected to a CUT&RUN assay to determine interaction of PML protein with HBV DNA using an anti-PML protein antibody (ab72137). DNA levels of enriched chromatin and input were determined using qPCR and calculated as percentage of input. The charts present data from two independent biological replicates measured in duplicate. A schematic representation of the HBV genome and the corresponding open reading frames is depicted below the graph. Additionally, the localization of CpG islands 1 to 3 and regions of HBV core protein (HBc)-occupied HBV DNA are shown based on reference 48.

The association of HBV DNA and PML-NBs was further determined with a CUT&RUN (cleavage under targets and release using nuclease) assay. Here, antibodies for the protein of interest direct an MNase nuclease to DNA interacting with the respective protein. Cleaved DNA can be released from digitonin-permeabilized cells by diffusion and analyzed by qPCR. As positive control for the CUT&RUN assay, binding of tri-methyl-histone H3 to HBV DNA was determined (Fig. S2). Here, binding of PML protein to several regions of nuclear HBV DNA, which is mostly cccDNA (48), was observed (Fig. 8). PML protein preferentially interacted with cccDNA regions around CpG island 3, which was also shown to be bound by HBV core (48) and in HBV core-occupied CpG island 2 (48), albeit to a lesser extent than in CpG island 3 (Fig. 8). PML protein additionally interacted with HBV DNA at CpG island 1 and in the pre-S1 region (Fig. 8), neither of which is in contact with HBV core (48). These findings further stress the importance of PML-NBs in the regulation of HBV cccDNA and substantiated the role of HBV core in bridging the interaction between HBV DNA and PML-NBs.

### Interference with efficient SUMO modification impairs core nuclear entry and HBV infection.

**FIG 9:**
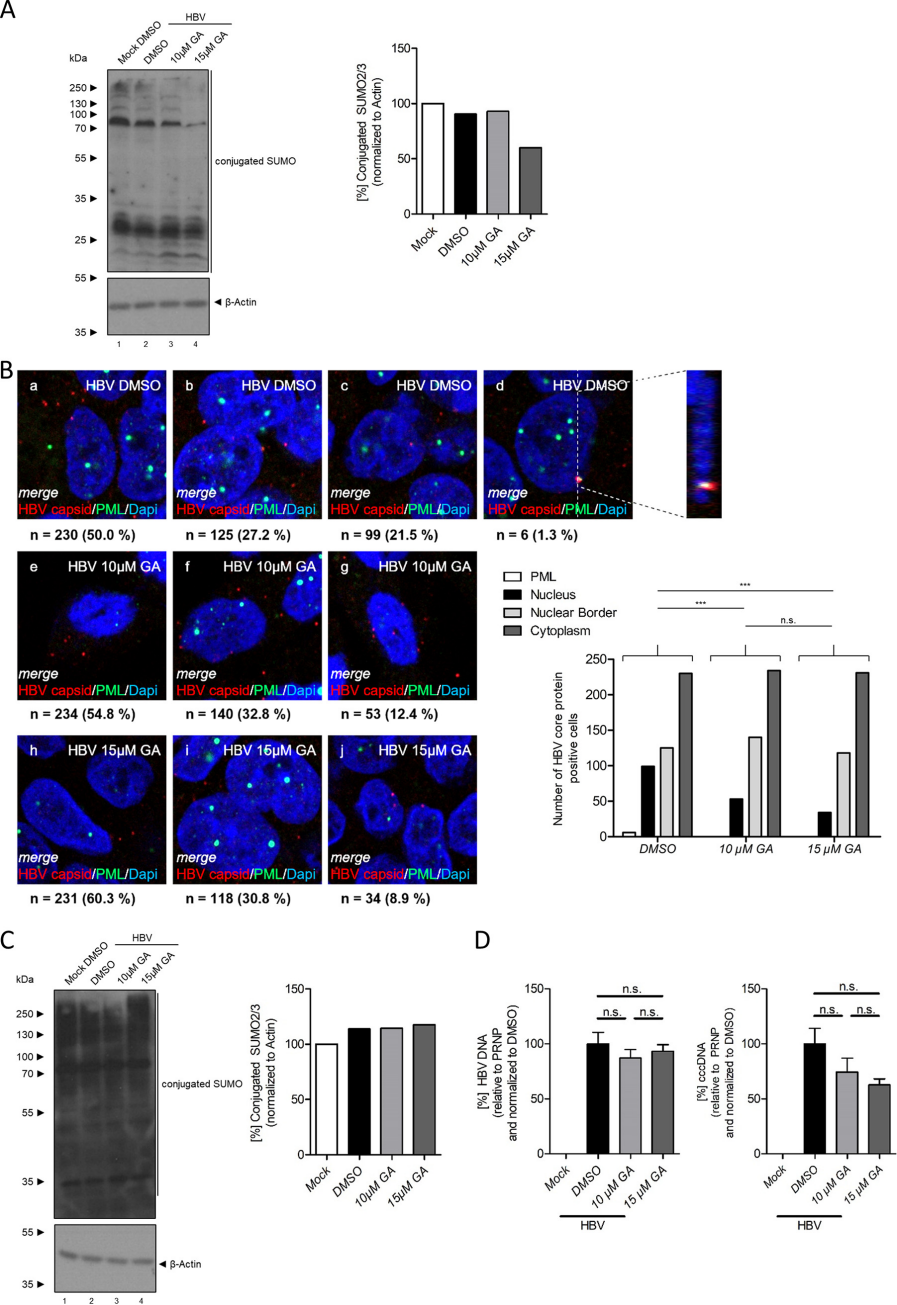
Interference with SUMO modification using ginkgolic acid serves as proof of concept. HepG2-NTCP cells were differentiated using 2.5% DMSO for 3 days. Eight hours prior to infection, cells were primed by addition of 10 or 15 μM GA. DMSO was used as the control. Cells were infected with HBV at an MOI of 200 in the presence of 10 or 15 μM GA and DMSO. (A) Cells were harvested 16 h postinfection (24 h posttreatment), and whole-cell protein lysates were prepared and analyzed by SDS-PAGE and Western blotting using anti-SUMO2/3 (ab81371) and anti-β-actin (AC-15) antibodies. Intensity of conjugated SUMO2/3 was determined using Fiji (version 1.45s) and normalized to mock treatment. (B) Cells were fixed with 4% PFA at 16 h postinfection (24 h posttreatment) and stained using anti-PML protein (sc-966) and anti-HBV capsid (Dako B0586) antibodies. Primary antibodies were detected using Alexa Fluor 488 (green; PML protein)- and Alexa Fluor 647 (red; HBV capsid)-conjugated secondary antibodies, and the nuclei were costained with DAPI. Pictures and Z-stacks were taken using a Zeiss LSM 980 laser scanning microscope. Representative merge images for at least 383 cells treated with DMSO (panels a to d) or with 10 (panels e to g) or 15 (panels h to j) μM GA are shown. (C) Cells were cultivated in medium without GA for 7 days, and whole-cell protein lysates were prepared and analyzed by SDS-PAGE and Western blotting using anti-SUMO2/3 (ab81371) and anti-β-actin (AC-15) antibodies. Intensity of conjugated SUMO2/3 was determined using Fiji (version 1.45s) and normalized to mock treatment. (D) Cells were cultivated in medium without GA for 7 days and total DNA was isolated. Total HBV DNA (right) and cccDNA (left) were determined by qPCR. The bar charts represent six independent experiments measured in triplicate. Statistically significant differences were determined using one-way ANOVA. n.s., not significant.

Taken together, the results presented above pinpointed the role of HBV core SUMO PTM in the nuclear entry and early events in cccDNA biogenesis directly after nuclear entry. As proof of concept that SUMO modification at early times is necessary for HBV nuclear entry and efficient cccDNA formation, the cellular SUMOylation machinery was inhibited using ginkgolic acid (GA). Ginkgolic acid globally interferes with SUMOylation by binding to the SUMO E1-activating enzyme and inhibiting E1-SUMO intermediate formation (49). Differentiated HepG2-NTCP-K7 cells were treated for a total of 24 h at concentrations which had been shown to interfere with efficient SUMO modification but had no effect on proliferation of HepG2 cells (50) (Fig. S3). Cells were primed by addition of 10 or 15 μM GA 8 h prior to infection, and GA was left on the cells during HBV infection. Inhibition of cellular SUMO modification was assessed by Western blotting and revealed a dose-dependent reduction in conjugated SUMO2/3 (Fig. 9A). After removal of the inoculum at 16 h postinfection (24 h posttreatment), HBV-infected cells with and without GA treatment were analyzed using immunofluorescence staining for incoming HBV capsids or capsid intermediates and PML protein. In the dimethyl sulfoxide (DMSO) control, the results shown above were further confirmed. The majority of incoming HBV capsids/capsid intermediates was observed to still reside in the cytoplasm (Fig. 9B, panel a), while 27.2% were found at the border of the nuclear DAPI staining (Fig. 9B, panel b), and 21.5% of incoming HBV particles had already been processed to the nucleus (Fig. 9B, panel c). A minor specific fraction of 1.3% of incoming HBV particles and HBV core was observed to localize to PML-NBs in untreated cells (Fig. 9B, panel d). Treatment with 10 and 15 μM GA gradually decreased the proportion of nuclear HBV capsid/capsid intermediate staining to 12.4% (Fig. 9B, panel g) and 8.9% (Fig. 9B, panel j), respectively. It is noteworthy that no localization of HBV capsids/capsid intermediates to nuclear PML-NBs could be observed during treatment with 10 or 15 μM GA (Fig. 9B), resulting in a significant decrease of HBV capsid nuclear entry in cells treated with GA (Fig. 9B). The effect of early GA application on HBV genome replication at later time points was then further investigated. Differentiated HepG2-NTCP-K7 cells were pretreated with 10 or 15 μM GA 8 h prior to infection, and GA was left on the cells during HBV infection. After 16 h, the inoculum and the GA were removed, and the cells were incubated in medium without compound for 6 days. After omission of GA for 6 days, the level of cellular SUMO modification had largely recovered (Fig. 9C). At the level of total HBV DNA, a minor decrease to 90% compared to the level in DMSO-treated cells was observed (Fig. 9D, left). In contrast, cccDNA levels were strongly and specifically reduced during GA treatment, with a reduction to 74.5% for 10 μM and 62.8% for 15 μM GA compared to DMSO treatment (Fig. 9D, right). This finding indicated that an efficient HBV infection and cccDNA generation did not appear, even after omission of GA for 6 days. Taken together, these results underlined the importance of SUMOylation in the entry process of HBV capsids and in early events during cccDNA formation.

## DISCUSSION

HBV infections are still a global health threat, although many humans are protected by an effective vaccination strategy. This is due to the pool of chronic infections and the lack of efficient therapies which target the HBV persistence reservoir, the cccDNA. HBV cccDNA is generated via a hepatocyte-mediated DDR and a cellular repair process of the entering incomplete rcDNA (1). Several of the DDR host factors involved were identified in recent years; however, the exact mechanism, many of the host proteins involved, and also the site of rcDNA-to-cccDNA conversion are still unclear (51).

Studies on PML-NBs proved a strong association of these nuclear complexes with the host DDR machinery. It was thus determined that PML-NBs are recruited to sites of DNA damage within the host genome and act as molecular hubs for the recruitment of DNA repair factors, which are often conjugated with SUMO proteins (22).

Here, we report that HBV core represents a novel player in the rcDNA-to-cccDNA conversion and that SUMO PTM and PML-NBs are host regulators of this pathogenic process to establish a persistent viral infection in human hepatocytes. Thus, we expand the panel of previous results on core-PML protein interaction (12) by proving SUMO PTM-dependent binding and colocalization of wt HBV core at PML-NBs during virus infection of differentiated hepatocytes. Additionally, we identified two HBV core fractions during infection: the unSUMOylated, soluble HBV core, which is probably involved in pgRNA encapsidation and assembly of progeny virions (48, 52, 53), and the SUMO-conjugated insoluble HBV core fraction, which is associated with the nuclear matrix of the infected cell with specific, but not all, PML-NBs inside the nucleus. HBV core is reported to bind to specific CpG islands within the HBV cccDNA and thereby promote active transcription from the cccDNA (48). Consequently, we propose a model wherein only HBV SUMO-core-bound viral DNA is recruited to specific cellular PML-NBs due to composition of associated host factors and SUMO/SIM interactions. HBV cccDNA molecules then reside juxtaposed to those multiprotein nuclear structures, which are involved in DNA repair, as observed by Chung and Tsai in HBV-producing HepG2 1.3ES2 cell line (12). This assumption is further supported by the findings of the CUT&RUN assay, where a stronger association of PML protein with HBV core-occupied CpG islands was observed, indicating that HBV core in a first step bridges the association of incoming viral DNA, with PML-NBs and core-independent binding appearing later in infection.

HBV core is known to be subject to extensive and dynamic PTM during the HBV life cycle, including highly dynamic phosphorylation, ubiquitinylation, and arginine methylation within the CTD (54). Our findings stress the importance of SUMOylation as a novel PTM in the functional regulation of HBV core. Analysis of HBV core SUMOylation by expression of the wt protein either alone or in hepatocytes with replicating HBV provided evidence that SUMO PTM occurs at HBV core lysine residues K7 and K96, which are highly conserved among all major HBV genotypes. Previously published data showed that protein binding and recruitment to PML-NBs is dependent on SUMO PTM (14, 38). Accordingly, an efficient block in HBV core SUMOylation by point mutation of SCM1 and SCM2 resulted in altered protein localization excluded from PML-NBs and no binding between the viral regulator and PML scaffold proteins.

As PML-NBs can be seen as model systems for phase separation in liquid-liquid interfaces, it is tempting to speculate that the specific PML-NBs which are able to recruit SUMO-modified HBV core harbor host factors necessary for cccDNA biogenesis. This is supported by previous studies on DDR factors involved in cccDNA formation showing that factors like FEN1 (23–26), DNA ligase 1 (3, 26, 27), and PCNA (28–35) are SUMO modified and associate with PML protein to promote their function. The PML-NB complexes having the capacity to support cccDNA formation might therefore be low in SUMO, to be able to recruit SUMO-modified HBV core protein together with the rcDNA on the one hand and SUMOylated DDR factors on the other hand. This hypothesis is further supported by the findings by Sengupta et al. showing that Sp110 is deSUMOylated by HBx and thereby excluded from PML-NBs (13), which are probably themselves low in SUMO.

Further experiments using pHBV1.1 with an HBV infection background showed a severe defect in nuclear entry when we analyzed the HBV core protein with mutations in SCM1 and SCM2, which possess an almost exclusively cytoplasmic phenotype. As SUMOylation of proteins is known to be a regulator of subcellular and especially nuclear localization (55), these data indicate that HBV core SUMOylation is a prerequisite not only for its association with PML-NBs but also for nuclear entry during infection *per se*. In line with this hypothesis, the *in silico* analysis of docking of SUMO2 to HBV core within a tetrameric core protein structure showed a steric overlap between the SUMO moiety and the adjacent HBV core protein subunit within the tetramer. This finding indicates a role for SUMOylation during HBV nucleocapsid disassembly, which we further confirmed by *in vitro* SUMOylation assays including purified, recombinantly expressed wt HBV nucleocapsids that were readily SUMOylated and disassembled after *in vitro* SUMO modification.

In line with the exclusive extranuclear phenotype of the Δ*scm1/2* HBV core expressed during infection, we did not observe efficient formation of a cccDNA pool after transfection of pHBV1.1 Δ*scm1/2* compared to the wt, indicating that viral capsids formed from pHBV1.1 Δ*scm1/2* are deficient in nuclear entry, and therefore, no delivery of rcDNA into the nucleus or generation of cccDNA from rcDNA can be detected. The determination of total HBV DNA and cccDNA in differentiated HepG2-NTCP-K7 cells infected with HBV wt or Δ*scm1/2* further confirmed this phenotype, showing a severe defect in total HBV DNA formation and a complete deficiency in cccDNA formation for the Δ*scm1/2* mutant viruses. Depletion of PML protein in HepaRG shPML cells resulted in a pHBV1.1 wt phenotype highly similar to that observed in pHBV1.1 Δ*scm1/2*, with almost no cccDNA formation, a finding which could be reproduced during HBV infection of HepG2 shPML cells, where a severe loss of total HBV DNA and cccDNA was observed. This can be explained by the role of PML protein and PML-NBs as molecular hubs for the host DNA repair machinery (22) and stresses the role of the SUMOylation-dependent HBV core recruitment to PML-NBs during HBV infection. The fact that coexpression of wt HBV core, but not of Δ*scm1/2* HBV core, could at least partially restore cccDNA formation from pHBV1.1 Δ*scm1/2* showed additionally that only a small proportion of SUMO-modified HBV core is sufficient to trigger HBV nucleocapsid disassembly and recruitment of rcDNA to PML-NBs as hubs for the host DNA repair and cccDNA conversion. It is noteworthy that we and others reported a strong association between protein SUMOylation and phosphorylation, with phosphorylation being a prerequisite for efficient SUMO modification (56–59). Based on these studies and our results, we suggest that SUMO modification is a trigger for disassembly of the HBV nucleocapsid within the nuclear basket of the nuclear pore complex. Previous studies showed that HBV core proteins in incoming, mature, rcDNA-containing nucleocapsids become rephosphorylated within the host cell and that this rephosphorylation is necessary for efficient targeting to the nuclear pore complex and augments cccDNA formation, while abrogation of phosphorylation in the N-terminal domain interferes with cccDNA formation (60–63). However, it was also shown that rephosphorylation *per se* is not sufficient to promote HBV capsid disassembly (60). We therefore propose that rephosphorylation of incoming mature HBV nucleocapsids is the prerequisite for subsequent SUMOylation and capsid disassembly at the nuclear pore complex. It is noteworthy that assembly of HBV nucleocapsids and encapsidation of the pgRNA results in the dephosphorylation of HBV core (64), which might prevent SUMO modification and therefore enable efficient assembly of mature rcDNA-containing nucleocapsids. HBV core SUMOylation might therefore act as a molecular switch for the function of HBV core within the HBV life cycle. This further explains the presence of two fractions of HBV core in infected hepatocytes: a SUMO-modified, nuclear matrix- and/or PML protein-associated fraction and a soluble, non-SUMOylated fraction, which is involved in nucleocapsid assembly and generation of progeny virions.

Intriguingly, Hong and Hu recently reported that lysine-to-arginine mutations at SCM1 and SCM2 in HBV core did not affect cccDNA formation in HepG2 and Huh7 cells and even increased cccDNA formation when normalized to rcDNA (65), a finding similar to that in another study in which a lysine-to-alanine mutation at SCM2 led to an increase in cccDNA formation (66). Our work, in contrast, showed that lysine-to-arginine mutations in SCM1 and SCM2 significantly reduced cccDNA formation normalized to total HBV DNA, which is mainly rcDNA, as this outnumbers cccDNA by a factor of 100 to 1,000 (45) in HepaRG cells. A major difference between our study and previous ones is that during mutation of SCM1 and SCM2, we observed no decrease in the levels of total HBV DNA and also did not observe a severe defect in the expression of HBV core or the formation and secretion of HBV nucleocapsids, as shown by Hong and Hu or Cui et al. (65, 66). Our results are, however, consistent with a previous study by Garcia et al., where in the presence of SCM1 and SCM2 mutation no defect in core expression, nucleocapsid formation, rcDNA packaging, and secretion of rcDNA-containing nucleocapsids was observed (67). The difference between our study and previous work might be due to the different cell lines which were used, as in the HepaRG cell line used here, transfection of pHBV1.1 Δ*scm1/2* resulted in wt-like levels of HBV core protein expression and nucleocapsid formation, and we could even detect secretion of DNA-containing virions. This main difference in the occurrence of markers for HBV rcDNA, to which the cccDNA values are normalized, might explain the different outcome of our study. It therefore seems that the protein levels of HBV core, HBV nucleocapsid formation, and secretion from HBV Δ*scm1/2* is better supported by HepaRG cell lines. We even isolated a notable amount of HBV virions from the supernatant of HepaRG cells transfected with pHBV1.1 Δ*scm1/2* which packaged rcDNA and could be used for infection in HepG2-NTCP cells, where the Δ*scm1/2* mutant virions showed a severe defect in cccDNA formation compared to the wt, further validating our present results.

**FIG 10:**
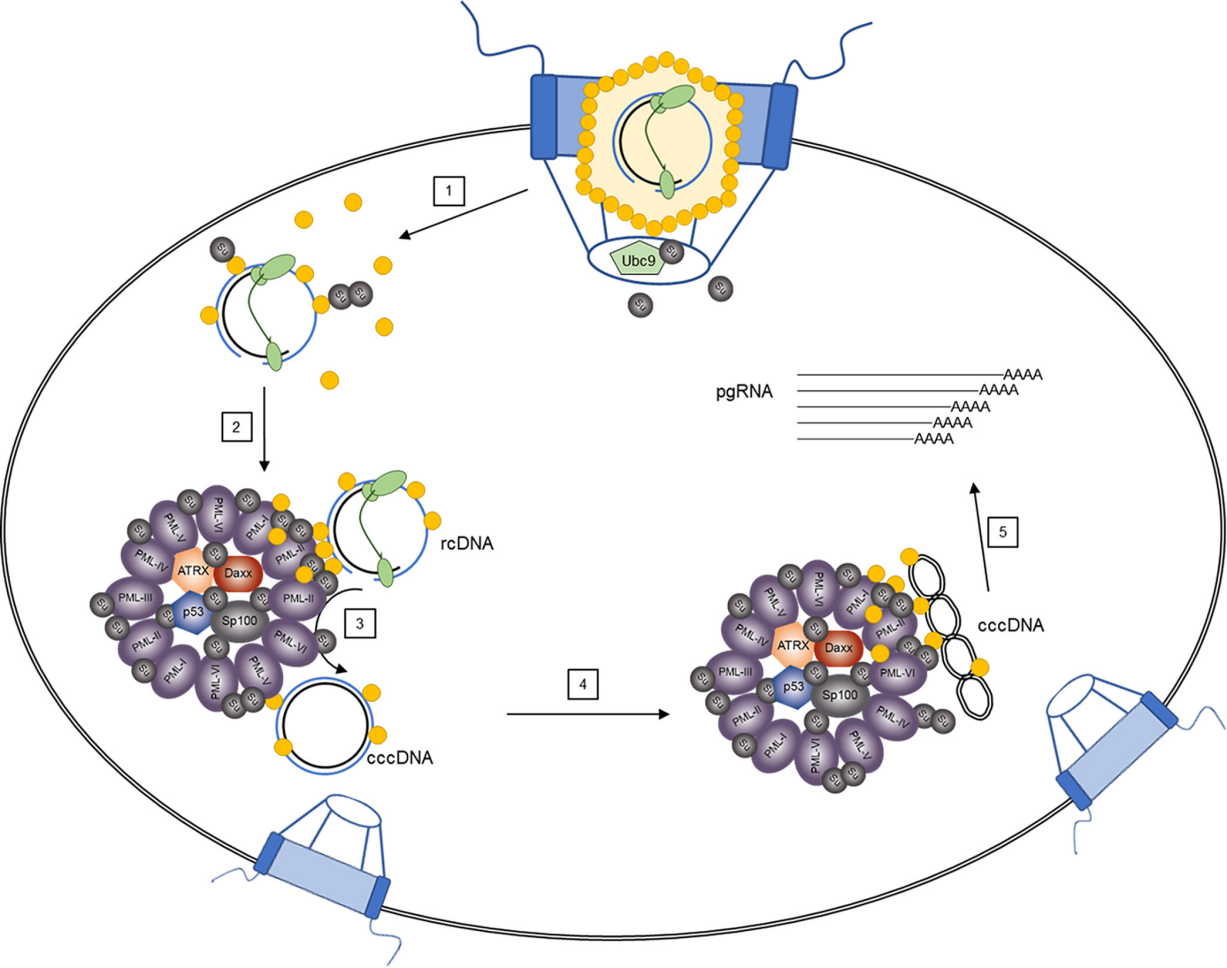
Proposed model of the impact of HBV core protein SUMOylation on HBV replication. Upon entry into the host cell, the nucleocapsid containing uncoated rcDNA is transported to the nuclear pore complex. Here, disassembly of the nucleocapsid is triggered by SUMOylation of HBV core protein monomers within the nucleocapsid (step 1). SUMOylated HBV core proteins that are still associated with the rcDNA are subsequently recruited into specific PML-NBs, probably via SUMO-SIM interactions (step 2), where the rcDNA-to-cccDNA conversion can take place (step 3), and the productive HBV replication cycle starts (steps 4 and 5).

In conclusion, our results provide novel insights into the molecular life cycle of HBV. We hypothesize that upon entry into the host cell, the rcDNA containing HBV nucleocapsid is transported to the nuclear pore complex. Within the nuclear basket of the nuclear pore complex, several HBV core subunits within the HBV nucleocapsids become SUMO modified by the host SUMOylation machinery, which has access to the nuclear basket (68). SUMOylation of HBV core then triggers disassembly of the HBV nucleocapsid, and the HBV DNA and the SUMOylated HBV core are corecruited into the nucleus to PML-NBs, where we suggest that repair of the rcDNA to cccDNA by the host DNA repair machinery takes place (Fig. 10).

Our work demonstrates a substantial interplay between PML-NBs, HBV core SUMOylation, and rcDNA-to-cccDNA conversion by the host cell DDR. Whole-scale inhibition of the cellular SUMOylation machinery using GA at early stages of HBV infection was therefore assumed to interfere with efficient HBV replication. After 24 h of treatment, GA induced a dose-dependent decrease in global cellular poly-SUMOylation associated with a significant decline in nuclear entry of HBV core and subsequent association with PML-NBs in cells primed by treatment with GA. The proportion of HBV core stalled at the border of the nuclear DAPI staining or stuck in the cytoplasm, however, increased during GA application. These findings provided further evidence for the role of HBV core SUMO PTM as the trigger for capsid disruption in the nuclear basket of the NPC and the subsequent nuclear entry and recruitment to PML-NBs. At 7 days postinfection, a dose-dependent decrease in cccDNA levels was observed. This indicated that HBV infection did not recover from the initial inhibition of nuclear entry and recruitment of HBV core together with the rcDNA to PML-NBs as the site of cccDNA formation. Altogether, these findings served as a proof of concept for our hypothesis that nuclear entry of HBV core protein and the rcDNA depends on HBV core SUMO PTM and that rcDNA-to-cccDNA conversion is governed by SUMOylation and PML-NBs. As inhibitors of several steps of the SUMO cascade are already in clinical trials or even FDA approved for other indications (69, 70), a repurposing of these drugs to treat HBV infection seems feasible.

We provide evidence for a profoundly important role of HBV core SUMOylation during *de novo* cccDNA formation. A similar impact on the intracellular cccDNA amplification pathway, which depends on delivery of progeny rcDNA to the nucleus by newly formed nucleocapsids, can be expected. Thus, inhibition of efficient SUMO conjugation to HBV core and tight association with PML protein and PML bodies might prove an innovative therapeutic strategy in the eradication of acute and chronic HBV infection.

## MATERIALS AND METHODS

### Cell culture and cell lines.

HepaRG cells (71) (Thermo Scientific) and HepaRG cells stably expressing His_6_-SUMO2 (HepaRG SUMO2) or His_6_-HA (HepaRG His/HA) (kindly provided by Ron Hay, University of Dundee, UK) were grown in Dulbecco’s modified Eagle medium (DMEM) (Sigma-Aldrich) supplemented with 10% fetal bovine serum (FBS) (Thermo Scientific), 100 U of penicillin and 100 μg of streptomycin (both from Thermo Scientific) per mL, 5 μg/mL bovine insulin, and 0.5 μM hydrocortisone (both from Sigma-Aldrich) at 37°C and 5% CO_2_. HepG2-NTCP-K7 cells (72) were cultured in DMEM supplemented with 10% FBS, 100 U of penicillin, 100 μg/mL of streptomycin, 2 mM l-glutamine (Thermo Scientific), and 1 mM nonessential amino acids (Thermo Scientific) and sodium pyruvate (Thermo Scientific) at 37°C and 5% CO_2_. Differentiation of HepG2-NTCP-K7 cells was induced by addition of 2.5% DMSO (SERWA) to the medium. All cell lines are frequently tested for mycoplasma contamination. PML protein depletion in HepaRG cells (HepaRG shPML) and HepG2-NTCP-K7 cells (HepG2 shPML) was mediated by lentiviral transduction of a validated PML protein shRNA targeting all isoforms of the PML protein (73). As respective controls, nontargeting, scrambled control shRNAs were used (HepaRG shCTL and HepG2 shCTL). Transduced cells were selected by addition of 2 μg/mL of puromycin (Thermo Scientific) to the corresponding culture medium.

For inhibition of SUMO modification, ginkgolic acid (C_15:1_; Sigma-Aldrich) was dissolved in DMSO to a stock concentration of 20 mM and stored at −20°C. Ginkgolic acid was used at concentrations of 10 and 15 μM for a total of 24 h. As a corresponding control, DMSO treatment was used. To assess the cytotoxicity of ginkgolic acid, HepG2-NTCP-K7 cells were treated with ginkgolic acid for 4 days at different concentrations up to 25 μM. Cytotoxicity was determined at day 4 using the Promega CellTiter-Blue cell viability assay according to the manufacturer’s manual. Fluorescence was measured using a GloMax Discover microplate reader.

### Plasmids and transient transfection.

The HBV core protein coding sequence (HBV D3; sequence ID CCK33732.1) was cloned into the pcDNA3.1-HA (pcDNA-HA) vector using BamHI and EcoRI. As a plasmid-based HBV replicon system, pHBV1.1 wt containing 1.1 copies of the HBV genome under the control of a HCMV promoter was used (74). Mutations of SCM1 and SCM2 were introduced by PCR using the following primers: ΔSCM1-fwd, TTATAGAGAATTTGGAGCTACTGTGGAG; ΔSCM1-rev, GGGTCGATGTCCATGAATTC; ΔSCM2-fwd, AGGTTCAGGCAACTCTTGTGG; ΔSCM2-rev, TAGGCCCATATTAGTGTTGAC. All primers were purchased from Metabion. Transient transfection of cells was performed as previously published using linear 25-kDa polyethyleneimine (Polysciences) (75).

### Viruses and HBV infection.

Genotype D HBV wt virus stocks were prepared from HepAD38 cells as previously described (46). Differentiated HepG2-NTCP-K7 cells were infected at the indicated multiplicities of infection (MOI) as previously described (72).

### Antibodies.

Primary antibodies specific for HBV proteins included anti-HBV core protein mouse monoclonal antibody (MAb) 8C9-11 (72), anti-HBV core protein mouse MAb sc-23945 (Santa Cruz), both recognizing linear epitopes within the HBV core protein, and HBV capsid/capsid-intermediate-specific rabbit polyclonal antibody (PAb) B0586 (Dako). Primary antibodies used for epitope tags and cellular targets included anti-HA rat MAb 3F10 (Core Facility for Monoclonal Antibodies, Helmholtz Zentrum München), mouse MAb against the His_6_ tag (Clontech), anti-PML protein mouse MAb sc-966 (Santa Cruz), polyclonal rabbit anti-PML protein PAb NB100-59787 (Novus Biologicals), polyclonal rabbit anti-PML protein ab72137 (Abcam), monoclonal mouse anti-SUMO2/3 ab81371 (Abcam), and anti-beta-actin mouse MAb AC-15 (Sigma-Aldrich). Secondary antibodies conjugated to Alexa Fluor 488 were purchased from Thermo Scientific, and secondary antibodies coupled to horseradish peroxidase or Alexa Fluor 647 were anti-rabbit IgG, anti-mouse IgG, anti-mouse light chain IgG, and anti-rat IgG (all from Dianova).

### Protein analysis.

Whole-cell protein lysates were prepared using radioimmunoprecipitation assay (RIPA) lysis buffer, and coimmunoprecipitation (co-IP) assays were performed basically as published elsewhere (75). Samples for coimmunoprecipitation were precleared using 30 μL Pansorbin (Millipore Calbiochem) per probe for 30 min. Per co-IP, 1.5 μL of HA rat MAb 3F10 (anti-HA) was coupled to 3 mg protein A-Sepharose beads (Sigma-Aldrich) for 1 h at 4°C before it was added to the precleared samples and rotated for 2 h at 4°C. Before elution, the beads were washed twice and eluted in 20 μL of 2× Laemmli buffer. Proteins were analyzed by SDS-PAGE followed by immunoblotting as previously described (75) using the PageRuler Plus prestained protein ladder (Thermo Scientific) to assign molecular weights. To verify SUMO modification of the HBV core protein and the lack of SUMOylation in the Δ*scm* mutant, HepaRG SUMO2 cells and HepaRG His/HA cells as controls were used, and protein lysates were prepared under denaturing conditions using guanidine hydrochloride. His_6_-SUMO2-modified proteins were purified as recently described (75). Proteins were analyzed by SDS-PAGE followed by immunoblotting as previously described (75). For quantitation of protein expression levels and degree of SUMO2 modification, Fiji software was used (76).

### Native agarose gel electrophoresis for determination of viral capsid formation.

For analysis of HBV capsid formation, samples were lysed in a low-stringency NP-40-based lysis buffer (50 mM Tris-Cl [pH 8.0], 100 mM NaCl, 1 mM EDTA, and 1% NP-40) on ice for 10 min. Nuclei and cell debris were pelleted by centrifugation at 12.000 × *g* and 4°C. Prior to loading on a 1.2% agarose gel, the samples were mixed with 6× loading buffer (50% glycerol and 0.1% bromophenol blue). For immunoblotting, the samples were transferred by capillary transfer using 10× saline sodium citrate (SSC; 1× SSC is 0.15 M NaCl plus 0.015 M sodium citrate) buffer on 0.2-μm nitrocellulose membranes (GE Healthcare). HBV capsids were visualized using the HBV capsid/capsid assembly intermediate rabbit PAb B0586. For determination of the amount of HBV core protein in the lysate, the same samples were denatured by addition of Laemmli buffer. Proteins were analyzed by SDS-PAGE followed by immunoblotting as previously described (75). Western blots were scanned and cropped using Adobe Photoshop CS5, and figures were prepared using Adobe Illustrator CS5 software. For quantitation of protein expression levels and degree of SUMO2 modification, Fiji software was used (76).

### Indirect immunofluorescence.

HepaRG and HepG2 cells were grown on cover slides for indirect immunofluorescence. At different times posttransfection or infection, the samples were fixed by incubation in 4% paraformaldehyde (PFA) for 10 min at room temperature. For HBV-infected HepG2 cells, an additional CSK washout was performed in indicated samples as recently described (42). Prior to blocking in Tris-buffered saline–BG (TBS-BG; BG is 5% [wt/vol] bovine serum albumin [BSA] and 5% [wt/vol] glycine) buffer for 30 min at room temperature, the samples were permeabilized in 0.5% Triton X-100 for 5 min. The cover slides were incubated with primary antibody diluted in phosphate-buffered saline (PBS) overnight at 4°C. Samples were washed three times with TBS-BG and incubated in the corresponding Alexa Fluor 488 (Invitrogen)- or Alexa Fluor 647 (Dianova)-coupled secondary antibodies. Prior to mounting in Mowiol, the slides were washed three times in TBS-BG. Samples were analyzed using a confocal laser-scanning microscope (Nikon TiE) and the Volocity software, a Zeiss LSM 980 laser scanning microscope and the Zeiss Zen 3 software, or a Zeiss Axio Observer Z.1 microscope and the Axiovision 4.8 software. To quantify colocalization, Spearman correlation ranks of the respective fluorescence channels were determined using Fiji (76) and the Pearson-Spearman correlation colocalization plugin (77).

### Quantitative PCR quantification of HBV DNA.

Total HBV DNA and HBV cccDNA were quantified by qPCR as previously described (72). For validation of the qPCR-mediated detection of cccDNA in the context of pHBV1.1 transfection, samples were codigested with DpnI and T5 exonuclease (both from New England Biolabs). Removal of plasmid background was monitored by agarose gel electrophoresis using a 1-kb DNA ladder to assign amplicon sizes (New England Biolabs).

### *In silico* prediction of SUMO conjugating motifs.

For the *in silico* prediction of SUMO conjugating motifs within the HBV core protein, the HBV core protein sequences were retrieved from the hepatitis B virus database HBVdb (78) and subjected to analysis using the software GPS-SUMO 1.0 (79). Alignments of HBV core protein sequences from different HBV genotypes were performed using ClustalOmega (80). To confirm conservation of SCM1 (K7) and SCM2 (K96) within HBV core from the different sequences, the frequency of lysine mutations at these sites was determined in HBVdb (78) and expressed as percent conservation.

### *In silico* docking of SUMO2 to HBV core protein.

For *in silico* docking of SUMO2 to the HBV core protein, the 3D structure files of the HBV core protein within the HBV nucleocapsid (PDB no. 3J2V_1) (81) and SUMO2 (PDB no. 2CHK_2) (82) were retrieved from the PDB and subjected to *in silico* docking studies using the ZDOCK server (83) using default settings and presuming interaction between the C-terminal double-glycine motif of SUMO2 and the respective SCM1 or SCM2 in HBV core. The predicted docking structures were visualized using the VMD (Visual Molecular Dynamics) program (84).

### *In vitro* SUMOylation assay.

For validation of a destabilizing impact of SUMO2 modification on HBV nucleocapsids, recombinant capsid-like particles (CLPs) from wt and Δ*scm1/2* HBV core protein were produced in Escherichia coli as previously described (85). In brief, expression from the T7 RNA polymerase promoter vector pRSF_T7-HBc183opt and its Δ*scm1/2* derivative in E. coli BL21*CP cells was induced by isopropyl-β-1-d-thiogalactopyranoside (IPTG), and then the cells were lysed in the presence of protease inhibitor cocktail (Roche) by treatment with lysozyme, Triton X-100, Benzonase and sonication. CLPs in the cleared lysate were enriched by sedimentation through sucrose gradients, all as detailed in reference 85. CLPs (200 nM) were assayed with an *in vitro* SUMOylation assay kit (Enzo Life Sciences) as recommended by the manufacturer. As a control, the samples were incubated without addition of SUMO1/2/3. Samples were analyzed by native agarose gel electrophoresis and Western blotting as described above.

### Isolation of HBV virions from supernatants of pHBV1.1-transfected cells.

HepaRG cells were grown in 100-mm dishes and transfected with 10 μg pHBV1.1 wt or pHBV1.1 Δ*scm1/2* 1 day postseeding. Supernatants of transfected cells were collected at days 3, 6, and 9 posttransfection. Cell debris was removed by centrifugation, and HBV virions were precipitated using 7.5% polyethylene glycol (PEG) and 0.5 M NaCl by overnight rotation at 4°C and subsequent centrifugation at 4,500 rpm. The pellet was resuspended in 300 μL PBS. The amount of isolated HBV virions was assessed using native agarose gel electrophoresis as described above.

### CUT&RUN assay for the determination of PML protein binding to HBV DNA.

HepG2-NTCP-K7 cells were infected with HBV at an MOI of 1,000 as described above. At 7 days postinfection, cells were harvested and subjected to the CUT&RUN assay (Cell Signaling Technologies) according to the manufacturer’s instructions. Polyclonal rabbit anti-PML protein ab72137 (0.6 μL; Abcam), 5 μL of negative-control rabbit (DA1E) MAb IgG XP isotype control (Cell Signaling Technologies), and 2 μL of tri-methyl-histone H3 (Lys4) (C42D8) rabbit MAb (Cell Signaling Technologies) were used as positive controls. Protein-bound HBV DNA was determined by qPCR as percentage of input according to the manufacturer’s recommendations, and the signal of the negative isotype control was subtracted.

All primers were purchased from Metabion (Table 1).

**TABLE 1:** Primers

Primer	Sequence
HBV ChIP 254-428 fwd	TCGTGGTGGACTTCTCTCAA
HBV ChIP 254-428 rev	TGAGGCATAGCAGCAGGAT
HBV ChIP 462-562 fwd	GTTGCCCGTTTGTCCTCTAATTC
HBV ChIP 462-562 rev	GGAGGGATACATAGAGGTTCCTTGA
HBV ChIP 1089-1154 fwd	GCTTTCACTTTCTCGCCAAC
HBV ChIP 1089-1154 rev	AACGGGGTAAAGGTTCAGGT
HBV ChIP 1305-1438 fwd	AGCAGGTCTGGAGCAAACAT
HBV ChIP 1305-1438 rev	GACGGGACGTAAACAAAGGA
HBV ChIP 1581-1693 fwd	GTGCACTTCGCTTCACCTCT
HBV ChIP 1581-1693 rev	GGTCGTTGACATTGCAGAGA
HBV ChIP 1901-2054 fwd	GCATGGACATCGACCCTTAT
HBV ChIP 1901-2054 rev	TGAGGTGAACAATGCTCAGG
HBV ChIP 2112-2297 fwd	CTGGGTGGGTGTTAATTTGG
HBV ChIP 2112-2297 rev	TAAGCTGGAGGAGTGCGAAT
HBV ChIP 2279-2392 fwd	TTCGCACTCCTCCAGCTTAT
HBV ChIP 2279-2392 rev	GAGGCGAGGGAGTTCTTCTT
HBV ChIP 2983-3133 fwd	ACAAGGTAGGAGCTGGAGCA
HBV ChIP 2983-3133 rev	GTAGGCTGCCTTCCTGTCTG

### Statistical analyses.

Statistical significance was assessed using a two-sided Welch’s *t* test or one-way analysis of variance (ANOVA) and the GraphPad Prism5 software.
