# Supplementary material for: SUMO Modification of Hepatitis B Virus Core Mediates Nuclear Entry, Promyelocytic Leukemia Nuclear Body Association, and Efficient Formation of Covalently Closed Circular DNA
Source: Microbiol Spectr. 2023 May 18;11(3):e00446-23. doi: 10.1128/spectrum.00446-23 (PMC10269885; doi:10.1128/spectrum.00446-23)
Supplement: Supplemental file 1 — Supplemental material. Download spectrum.00446-23-s0001.pdf, PDF file, 0.5 MB [file spectrum.00446-23-s0001.pdf]

SUPPLEMENTARY FIGURE 1 Hofmann *et al.*
(concerns figure 4)

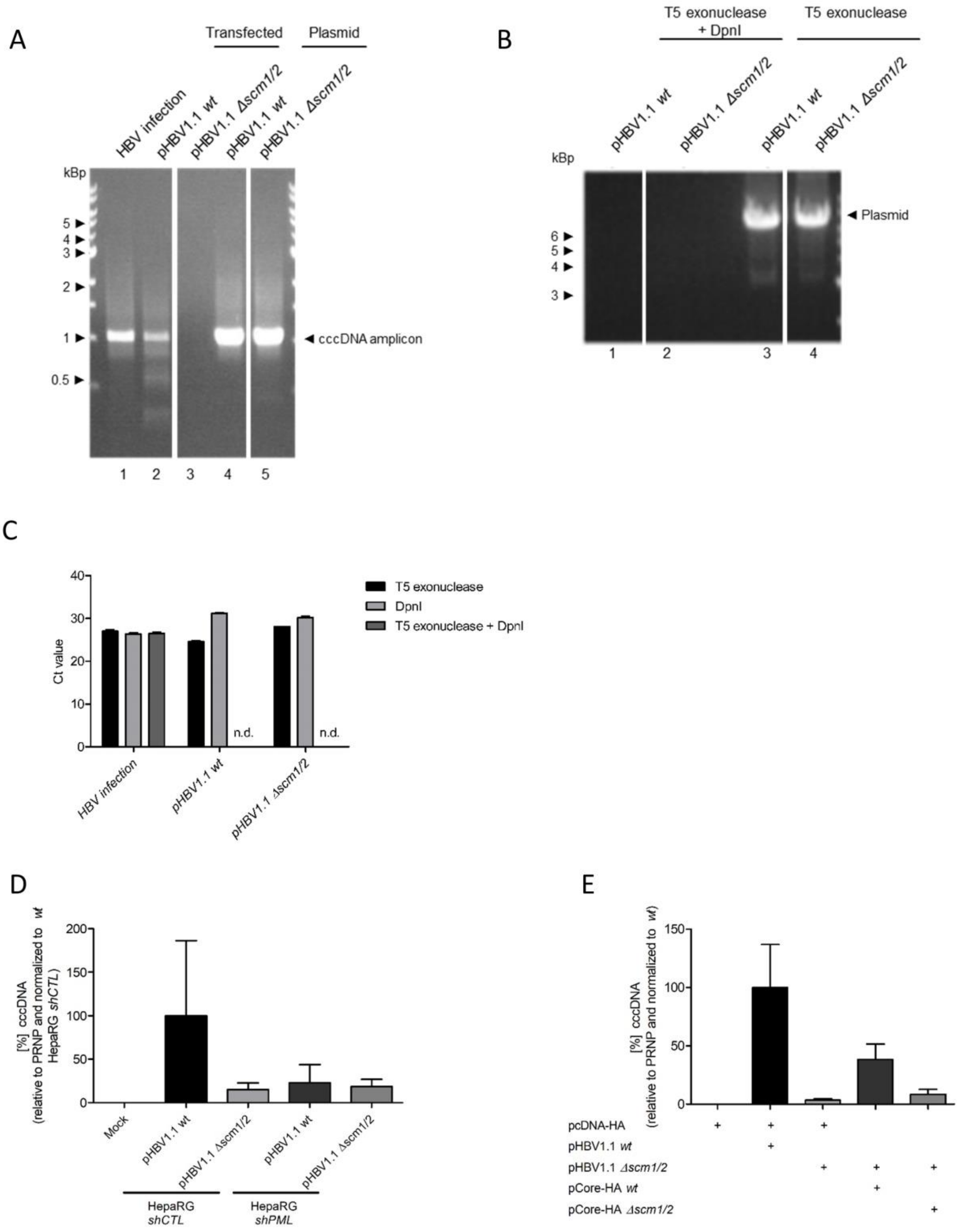

Supplementary figure 1: Validation of cccDNA quantification by qPCR in pHBV1.1 transfected cells. (A) cccDNA specific qPCR was performed with samples obtained from HBV infected HepG2-NTCP-K7 cells (lane 1), pHBV1.1 *wt* (lane 2) and Δ*scm1/2* (lane 3) transfected HepaRG cells and pHBV1.1 *wt* (lane 4) and Δ*scm1/2* (lane 5) plasmid alone. The resulting DNA was analysed using agarose gelelectrophoresis. **(B)** 500ng of pHBV1.1 *wt* (lanes 1 and 3) and Δ*scm1/2* (lanes 2 and 4) were diluted in DNA isolated from HepG2-NTCP-K7 cells using the MacheryNagel NucleoSpin Tissue Kit. Samples were either co-digested with T5 exonuclease and DpnI (lanes 1 and 2) or with T5 exonuclease alone (lanes 3 and 4). **(C)** DNA isolated from HBV infected HepG2-NTCP-K7 cells at 7d post infection or pHBV1.1 *wt* and Δ*scm1/2* plasmid DNA diluted in DNA isolated from HepG2-NTCP-K7 cells were either digested with T5 exonuclease alone, DpnI alone or co-digested with T5 exonuclease and DpnI and subjected to cccDNA specific qPCR. **(D)** HepaRG cells with stable transduction of a scrambled control shRNA (HepaRG *shCTL*), or a shRNA against a conserved region in PML (HepaRG *shPML*) were transfected with 3µg pHBV1.1 *wt* or Δ*scm1/2*. Total DNA of the samples was isolated, co-digested with T5 exonuclease and DpnI and cccDNA was measured 7 days post transfection by qPCR relative to *PRNP*. Bar charts represent mean values and standard deviations of three independent experiments measured in triplicates. **(E)** HepaRG cells were transfected with 3µg of pHBV1.1 *wt* or Δ*scm1/2* and 1µg of pcDNA-HA, pCore-HA *wt* or pCore-HA Δ*scm1/2*. 7days post transfection, total DNA was extracted, co-digested with T5 exonuclease and DpnI and subjected to qPCR for cccDNA. Bar charts represent mean values and standard deviations of three independent experiments measured in duplicates.

SUPPLEMENTARY FIGURE 2 Hofmann *et al.*
(concerns figure 8)

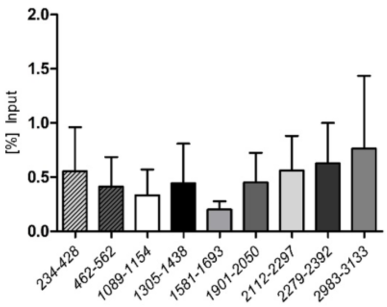

Supplementary figure 2: Tri-Methyl-Histone H3 binding to HBV DNA serves as positive control for Cut&Run assay. HepG2-NTCP-K7 cells were differentiated by addition of 2.5% DMSO for 3d. Cells were infected with HBV at a MOI of 1000. After 7d post infection, the cells were harvested and subjected to Cut&Run assay kit to determine interaction of Tri-Methyl-Histone H3 with HBV DNA using an anti- Tri-Methyl-Histone H3 (Lys4) (C42D8) Rabbit mAb antibody. DNA levels of enriched chromatin and input were determined using qPCR and calculated as [%] input. Bar charts represent data from two independent biological replicates measured in duplicates.

SUPPLEMENTARY FIGURE 3 Hofmann *et al.*
(concerns figure 9)

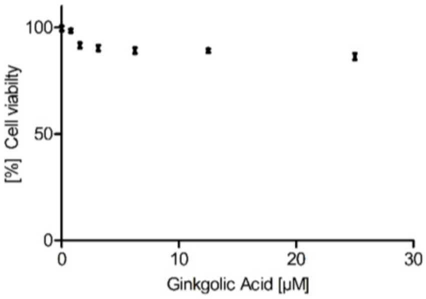

Supplementary figure 3: Ginkgolic acid treatment up to 25 µM does not interfere with cell viability of HepG2-NTCP-K7 cells. HepG2-NTCP-K7 cells were differentiated in presence of 2.5% DMSO for 3 days and then treated with ginkgolic acid at concentrations up to 25 µM. After 4 days, cell viability was assessed using the Promega CellTiter-Blue cell viability assay and normalized to the DMSO treated control.
